# Biophysical modeling of the whole-cell dynamics of *C*. *elegans* motor and interneurons families

**DOI:** 10.1371/journal.pone.0298105

**Published:** 2024-03-29

**Authors:** Martina Nicoletti, Letizia Chiodo, Alessandro Loppini, Qiang Liu, Viola Folli, Giancarlo Ruocco, Simonetta Filippi

**Affiliations:** 1 Department of Engineering, Università Campus Bio-Medico di Roma, Rome, Italy; 2 Center for Life Nano- & Neuro-Science (CLN2S@Sapienza), Istituto Italiano di Tecnologia, Rome, Italy; 3 Department of Medicine and Surgery, Università Campus Bio-Medico di Roma, Rome, Italy; 4 Department of Neuroscience, City University of Hong Kong, Hong Kong, China; 5 D-tails s.r.l., Rome, Italy; 6 Istituto Nazionale di Ottica del Consiglio Nazionale delle Ricerche (CNR-INO), Florence, Italy; 7 ICRANet—International Center for Relativistic Astrophysics Network, Pescara, Italy; Georgia State University, UNITED STATES

## Abstract

The nematode *Caenorhabditis elegans* is a widely used model organism for neuroscience. Although its nervous system has been fully reconstructed, the physiological bases of single-neuron functioning are still poorly explored. Recently, many efforts have been dedicated to measuring signals from *C*. *elegans* neurons, revealing a rich repertoire of dynamics, including bistable responses, graded responses, and action potentials. Still, biophysical models able to reproduce such a broad range of electrical responses lack. Realistic electrophysiological descriptions started to be developed only recently, merging gene expression data with electrophysiological recordings, but with a large variety of cells yet to be modeled. In this work, we contribute to filling this gap by providing biophysically accurate models of six classes of *C*. *elegans* neurons, the AIY, RIM, and AVA interneurons, and the VA, VB, and VD motor neurons. We test our models by comparing computational and experimental time series and simulate knockout neurons, to identify the biophysical mechanisms at the basis of inter and motor neuron functioning. Our models represent a step forward toward the modeling of *C*. *elegans* neuronal networks and virtual experiments on the nematode nervous system.

## Introduction

Modeling neurons and neuron networks is a powerful tool for understanding and predicting the information processing in the brain. The study of the relation between physical/chemical connections and signaling is challenging because of the complexity of the activated molecular pathways and the nature of the network itself.

A minimal, still complete, model for brain functioning, including all the essential living functions based on multiple perception mechanisms, as motion, food search, escape capabilities, and mate search, is provided by the *C*. *elegans* nervous system. Its whole brain, consisting of slightly more than 300 neurons, has been spatially mapped [1–3]. Physical connections (almost 9000 chemical synapses and gap junctions) among neurons are known, and the nature of some among the connections has been characterized [2, 4–10].

Despite the relative simplicity of the nematode brain, only a few mechanisms and sub-networks have been so far experimentally explored, including chemosensory [10–14], thermosensory [15, 16], and mechanosensory [9, 17] circuits.

Several computational works have successfully described the *C*. *elegans* whole brain or sub-circuits functioning with mathematical models [14, 18–25]. However, the focus of these works is the study of network dynamics. For this reason, in network simulations, single neurons are modeled with simplified equations, which do not consider the repertoire of dynamics observed in *C*. *elegans* and especially their physiological origin [26–32].

Nevertheless, it is also important to dissect the physiological mechanisms underlying the behavior of single neurons to elucidate the functioning of the nematode brain. In this context, detailed biophysical models might help to interpret experimental data, predict responses to different kinds of stimuli (e.g. current, voltage or chemical stimulations), and drive targeted experiments on *C*. *elegans* neurobiology, for example, by suggesting mutations or identifying molecular pathways of interest. To the best of our knowledge, few works have focused on this peculiar aspect of the *C*. *elegans* nervous system *in silico* investigation [30, 33–36]. The major limitations of biophysically accurate models are i) the need for refined electrophysiology data to identify parameters and ii) the computational cost, due to the high number of equations needed to adequately describe the dynamics of single ionic currents. These limitations are, nevertheless, overcome by their potential to explain the observed neuronal dynamics and their predictive potential to identify mechanisms and misfunctioning [30, 33, 34, 36–38].

In this paper, we model the electrical dynamics at the single neuron level of three interneurons, AVA, AIY, and RIM (which also acts as motor neuron), and three motor neurons, VA5, VB6, and VD5. The choice of the neurons is based on the availability of high-quality electrophysiology data [29, 30, 32, 39, 40] and the interesting variety of whole-cell dynamics exhibited by each of the selected neurons. These dynamics are not only interesting from a theoretical point of view, but also have important implications for understanding the functional role of these interneurons and motor neurons in neural circuits. Therefore, capturing the electrical dynamics of these neurons through computational modeling is crucial for gaining a more comprehensive understanding of their function. To model the six neurons, we rely on a set of ion currents, already used to model AWC and RMD [35–37] neurons, that we further enriched, including EXP-2, UNC-103, and KQT-1 currents [41–45]. The selection of ion currents included in each model is based on their gene expression profiles and on the availability of single channel experimental electrophysiology data to fit the model.

Main general features of the modelled neurons are reported below.

AVA are fundamental interneurons involved in the backward movement subcircuit. In particular, they participate in the coordination of motor responses to chemical [13, 46, 47] and mechanical [48, 49] stimuli promoting reversals. Recent results suggest AVA could be hub neurons, where sensory inputs from threat and reward sensory modalities and motor information from D-MNs are integrated [29]. Patch-clamp recordings on AVA neurons reported a depolarized resting membrane potential and a near-linear behavior in voltage-clamp experiments [29, 50] that might be related to K2P channels [50].

AIY are first-layer interneurons strongly involved in processing sensory information from olfactory, gustatory, and thermosensory neurons [12, 51–53]. They are postsynaptic to both olfactory and gustatory sensory neurons. AIY neurons are inhibited when AWC is activated by odor removal, working in combination with AIB in controlling the response to odor and food exposure [12, 54]. They are involved in suppressing turns and reversals, while enhancing smooth forward movements and dispersal [12, 51]. In patch-clamp experiments, AIY neurons show large non-inactivating and small inward currents, which confer to the neuron a pronounced ability to respond to hyperpolarizing stimuli [30].

RIM are second-layer interneurons that collect information from the internal animal state and external environment and integrate them to regulate the animal behavior. They act both as interneurons and motor neurons forming neuromuscular junctions with neck muscles [1]. RIM plays a double role, promoting and suppressing locomotion via the excitation and inhibition induced by electrical and chemical neurotransmission, respectively [55]. Moreover, with AVA and AIB, they belong to the olfactory circuit downstream of AWC olfactory neurons, where they are critical in regulating the AIB responses to odor [46]. Electrophysiological recordings classified RIM interneurons as "transient outward rectifying" neurons that smoothly hyperpolarize and depolarize under-current clamp [30].

VA5, VB6, and VD5 are ventral motor neurons involved in locomotion and innervating the ventral body muscles. VA and VB are excitatory cholinergic motor neurons regulating backward and forward locomotion, respectively [9, 56]. VB motor neurons also activate the inhibitory GABA-ergic D motor neurons and are involved in *C*. *elegans* proprioception [56]. VA5, VB6, and VD5 motor neurons show similar electrophysiological properties displaying large non-inactivating outward currents driven by SLO-2 channels [32, 40, 57]. The three motor neurons could be classified as "outward rectifying" neurons.

In this work, we propose detailed biophysical models reproducing the experimental current and voltage clamp recordings of the six selected neurons [29, 30, 32, 39]. In addition, we characterize the behavior of noticeable knockout (KO) cases, mimicking the action of pharmacological blockers. We also discuss our results in light of experimental data not used for parameter estimation and other computational works on the selected neurons [34, 38].

## Materials and methods

In this section, we briefly describe the electrophysiological properties of *C*. *elegans* neurons, the general mathematical model of the neurons, and how the six models have been implemented and optimized.

### *C*. *elegans* neurons electrophysiology and experimental data

In this section we introduce basic notions on *C*. *elegans* neurons electrophysiological properties. Since the first electrophysiological recording on *C elegans* neurons, performed by Goodman *et al*. [58], many works have highlighted a rich repertoire of neuronal dynamics in *C*. *elegans*, including regenerative responses, bistable responses, action potentials, and graded responses [26–33, 39, 58]. Such responses are originated by an interplay of voltage-gated potassium and calcium currents, since the nematode lacks the voltage-gated sodium channels, involved in mammalian action potentials.

In this study, in particular, we investigate interneurons and motor neurons that mainly show graded responses. To note that the action potential, that is a common feature of most excitable cells, has been observed and modelled so far only for AWA and AVL in *C*. *elegans* [30, 33], while it has not been observed in the neurons here investigated.

In the following we report a classification, based on the ionic selectivity, of the *C*.*elegans* ionic currents modelled by the authors in the present and in a previous study [36].

- Voltage-gated calcium currents. The three voltage-gated calcium currents of *C*. *elegans*: EGL19, UNC2, and CCA1 are representative of L-type, P/Q-type, and T-type currents, respectively.
- Voltage-gated potassium currents. This class represents the most numerous group of the modelled currents. We model transient (SHL1, SHK1, KVS1), non-inactivating (EGL2, EGL36, KQT1, KQT3), and inward rectifier currents (IRK, EXP-2, UNC103).
- Calcium-regulated potassium currents. The regulation played by calcium can be modelled in different ways, depending on the molecular mechanisms. The small-conductance (SK) current, driven by KCNL-1/4 channels, depends solely on the intracellular calcium concentration, while the big-conductance (BK) currents [59], driven by SLO-1 and SLO-2 channels, show a double regulation by intracellular calcium and membrane voltage [32, 60– 62]. In the biophysical models of RMD and AWC neurons [35– 37, 63], we modelled the SLO1 and SLO2 currents exclusively in the case of 1:1 stoichiometry with CaV (UNC-2 and EGL-19) channels. Here, we also implemented the model of isolated SLO1 and SLO2 currents, as described in [64] and detailed in the S1 File. Moreover, to ensure the proper coupling of the isolated BK channels dynamics with intracellular calcium, we adopted the model of intracellular calcium dynamics developed by Raman I. M. *et al*. [65, 66]. For a brief description of this model, we refer the reader to **S1 File**.

The neuron models presented in this work rely on experimental data available in literature [29, 30, 32, 40]. All the reference experimental recordings have been recorded from immobilized worms in the whole-cell configuration, using both voltage- and current-clamp protocols. In the case of VA5, VB6, and VD5 neurons, the reference paper reports, in addition to WT recordings, the recordings on mutants for some of the voltage-gated calcium and potassium currents [32]. Unfortunately, for the other neurons, only the whole-cell recordings in WT worms without any pharmacological blocking were available. For specific details on the experimental procedures, we refer the reader to the corresponding papers: [30] for RIM and AIY; [29] for AVAL and AVAR; [32, 40] for VA5, VB6, and VD5 (for further details on experimental procedures see also the S1 File).

To obtain a biophysical representation of the neuronal dynamics, for each neuron, we create the most possible complete list of expressed ionic channels (see **Table 1**). We used the gene expression profile from the CeNGEN database [67], we combined it with the profile available in the WORMBASE database [68], and further refined the profile with relevant literature [26, 30, 31, 50]. Overall, a set of seventeen ionic currents is used to model the six neurons. We also provide a new model for both SHL-1 and SHK-1 currents, relying on the experimental data from [28, 30, 44, 45]

**Table 1:** Gene expression profiles in modeled neurons. This table lists the modeled ionic currents and their expression profiles in the selected neurons. For all the neurons except VD5, the expression profiles were obtained from the CENGen database [67], using as threshold 1 for AIY, and 2 for AVA and RIM. In the case of VA5, VB6 and VD5, instead of referring to CENGEN, we selected the currents based on the electrophysiological characterization shown in [32]. The currents modelled for the first time in this work are highlighted in bold.

Channel gene	Mammalian ortholog	Ion selectivity	AIY	AVAL	AVAR	RIM	VA5	VB6	VD5
*shl-1*	*Shal*	*K* ^*+*^	✔	**×**	**×**	✔	**×**	✔	**×**
*shk-1*	*Shaker*	*K* ^*+*^	**×**	**×**	**×**	**×**	✔	✔	✔
*kvs-1*	*Kvs-1*	*K* ^*+*^	**×**	**×**	**×**	**×**	✔	✔	**×**
*egl-2*	*Eag*	*K* ^*+*^	**×**	✔	✔	✔	✔	**×**	**×**
*egl-36*	*Shaw*	*K* ^*+*^	**×**	**×**	**×**	✔	✔	**×**	**×**
***kqt-1***	*Kcnq*	*K* ^*+*^		**×**	**×**	**×**	**×**	**×**	**×**
*kqt-3*	*Kcnq*	*K* ^*+*^	**×**	**×**	**×**	✔	**×**	**×**	**×**
***exp-2***	*Kcnf*	*K* ^*+*^	**×**	**×**	**×**	**×**	**×**	✔	**×**
*irk-1/3*	*Kcnj*	*K* ^*+*^	✔	✔	✔	✔	✔	✔	✔
***unc-103***	*Kcnh*	*K* ^*+*^	**×**	✔	✔	✔	**×**	**×**	**×**
*slo-1*	*Slo*	*K* ^*+*^	✔	✔	✔	✔	✔	✔	✔
*slo-2*	*Slo*	*K* ^*+*^	✔	✔	✔	✔	✔	✔	✔
*kcnl-1/4*	*Kcnn*	*K* ^*+*^	✔	✔	✔	✔	✔	✔	**×**
*cca-1*	*Cav*, *T-type*	*Ca* ^*2+*^	**×**	✔	✔	✔	✔	✔	✔
*unc-2*	*CaV- P/Q-Type*	*Ca* ^*2+*^	✔	✔	✔	✔	✔	✔	✔
*egl-19*	*CaV*, *L-type*	*Ca* ^*2+*^	✔	✔	✔	✔	✔	✔	✔
*nca-1/2*	*Nalcn*	*Na* ^*+*^	✔	✔	✔	✔	✔	✔	✔

### Neurons modeling

Our models are based on the Hodgkin-Huxley model modified to reproduce the *C*. *elegans* neuronal dynamics [69]. Briefly, the membrane voltage dynamics of a neuron is described by the classical equation of the Hodgkin-Huxley (HH) model:

$$C\frac{dV}{dt}=-I_{ion}+I_{stim}$$ 1

where *C* is the membrane capacitance, and *I*_*stim*_ is the external current applied to the neuron to elicit the responses in the current-clamp configuration. The term *I*_*ion*_ represents the total ionic current in the cell, including contributions from potassium, calcium, calcium-regulated, and leakage currents:

$$I_{ion}=I_{K}+I_{Ca}+I_{K-Ca}+I_{leak}$$ 2

Each term in the right side of Eq 2 represents the total current of potassium or calcium ions which could be itself the sum of many different currents associated with the diverse kinds of ionic channels expressed in the cell.

Each ionic current has been modelled adapting the classical Hodgkin-Huxley model to reproduce the main ionic currents of the nematode. Briefly, the *x-th* ionic current has been modelled according to the Hodgkin-Huxley formalism as follows:

$$I_{X}={\bar{g}}_{x}∙m_{x}^{p}∙h_{x}^{q}∙(V-E_{rev})$$ 3

where ${\bar{g}}_{x}$ is the maximal conductance, and *E*_*rev*_ is the reversal potential of the ionic species: -80 mV for K^+^, and 60 mV for Ca^2+^. $m_{x}^{p}$ and $h_{x}^{q}$ represent the activation and the inactivation variables, respectively:

$$\frac{dm_{x}}{dt}=\frac{m_{x,\infty }-m_{x}}{\tau_{x,m}}$$ 4

$$\frac{dh_{x}}{dt}=\frac{h_{x,\infty }-h_{x}}{\tau_{x,h}},$$ 5

where, *m*_*x*,∞_ and *h*_*x*,∞_ represent the steady state values of the activation and inactivation variables, and *τ*_*x*,*h*_ and *τ*_*x*,*m*_ are the activation and inactivation time constants. For the full list of equations and parameters of single-currents models we refer the reader to the **S1 File** and to [36];

In addition to the currents mentioned above for each neuron, we added a leakage current to take into account other currents not explicitly modeled:

$$I_{leak}={\bar{g}}_{leak}(v-E_{rev})$$ 6

where ${\bar{g}}_{leak}$ is the maximal conductance and *E*_*rev*_ is the reversal potential.

All the model presented in this work have been developed in the single-compartment approximation, in which the neuron is modeled as a cylindrical compartment whose surface is equivalent to the total surface of the cell, whose value is obtained from the Neuromorpho database (https://neuromorpho.org/). We adopted this approximation because of the limited information available on the specific distribution of the ionic channels in the different regions of the neuron. Moreover, there are few works focused on studying the different functionalities of the neuronal compartments in *C*. *elegans*, and these works are not specifically focused on their electrical behavior or on the neurons considered in this work [27, 54, 70, 71]. However, despite its well-known limitations, this approach has already been successfully applied for modeling *C*. *elegans* neurons such as AWA, AIY, RIM, AVL, AIA, and AFD [30, 33, 34].

#### Model implementation and optimization

In this section, we describe how we implemented and optimized the models of the six neurons. The models of the ionic currents and whole neurons are implemented in NEURON [72, 73] and solved in Python. For each neuron, the parameters describing the activation and inactivation (and the corresponding time constants) of the ionic currents were used as fixed parameters, while the conductance values, representing the relative weights of the currents, were used as free parameters in the optimization procedure. Moreover, in the optimization procedure, we adjusted the reversal potential of the leakage current and the membrane capacitance. To obtain the optimal set of parameters, we used a hybrid optimization strategy that combines evolutionary computation [73], using the Python library Inspyred (https://pypi.org/project/inspyred/), and least square minimization of SciPy [74]. During the optimization procedure the HH equations are solved with NEURON.

Both the evolutionary algorithms and the least square minimization are based on the minimization of the root mean distance between the experimental and the simulated data using one or a combination of the following fitness functions:

$$\psi_{voltage-clamp}=\sqrt{\frac{\sum_{i=1}^{M}\sum_{j=1}^{N}{\left(I_{EXP}^{ij}-I_{SIM}^{ij}\right)}^{2}}{MN}}$$ 7

$$\psi_{current-clamp}=\sqrt{\frac{\sum_{i=1}^{M}\sum_{j=1}^{N}{\left(V_{EXP}^{ij}-V_{SIM}^{ij}\right)}^{2}}{MN}}$$ 8

$$\psi_{IV-curve}=\sqrt{\sum_{i=1}^{M}{\left(V_{EXP}^{i}-V_{SIM}^{i}\right)}^{2}}$$ 9

where M represents the number of current\voltage steps, N is the number of points in the experimental recording, *V*_*SIM*_ and *I*_*SIM*_ are the simulated voltage and current, and *V*_*EXP*_ and *I*_*EXP*_ are the corresponding experimental data. The fitness function used in the optimization problem was selected based on available experimental data for the neurons considered.

In **Table 2** we summarize the optimization procedure followed for each of the selected neurons. It has to be noted that for VA5, VB6, and VD5, we derived the set of parameters by a careful fine-tuning of the conductances based on the complete electrophysiological characterization shown in [32]; while, for the neurons whose electrophysiological characterization is not known, we applied either a least square minimization (in AVAL and AVAR) or a hybrid optimization based on multiobjective optimization with Nondominated Sorting Genetic Algorithm (NSGA-II) and least square minimization. In the NSGA minimization, we used both the current and voltage-clamp recordings.

**Table 2:** Optimization of the neurons. In the table, we list the information relative to the optimization procedure for each modelled neuron. The second column reports the optimization algorithm, while in the third, fourth, and fifth columns, we provide information on the type of experimental data, the fitness function, and the reference literature, respectively.

Neuron	Optimization procedure	Experimental data type	Fitness function	Reference paper
AIY	Least square minimization	Current-clamp	Eq 7	[30] Current-clamp recording is available in the supporting
RIM	Evolutionary computation + least square minimization	Voltage and Current-clamp	Eq 8	[30] Current and voltage-clamp recordings are available in the supporting
AVAL	Least square minimization	Current-clamp	Eq 8	[29] Current-clamp recording provided by the authors of [29]
AVAR	Least square minimization	Current-clamp	Eq 8	[29] Current-clamp recording provided by the authors of [29]
VA5	Hand-tuning based on currents dissection	Voltage-clamp	Eq 7	[40] Voltage-clamp available in the supporting
VB6	Hand-tuning based on currents dissection	Steady-state I-V curve	Eq 9	[32] I-V curve extracted from Fig 1
VD5	Hand-tuning based on currents dissection	Steady-state I-V curve	Eq 9	[32] I-V curve extracted from Fig 1

For each neuron, we obtained multiple sets of parameters reproducing the behavior of the neuron. Among the different sets of parameters, we selected the one that best reproduced the current and voltage-clamp characteristics.

In

**Table 3** we provide the selected set of parameters for each neuron. In particular, we report the set of conductances, the reversal potential of the leakage current, and the membrane capacitance.

**Table 3:** List of channels included in single neuron models. The modelled channels are listed based on the encoding *C*.*elegans* gene and their ion selectivity (first and third columns). For each neuron, the ionic currents included in the model are listed with the corresponding value of the maximal conductance. The symbol "-" indicates that the current is not included in the set of channels used in the model and/or the corresponding channel is not expressed in the neuron. The two values of *slo-2* conductance in VA5, VB6, and VD5 neurons represent the isolated and coupled (*slo-2*:*egl-19*) conductance, respectively. The reversal potential for K^+^ and Ca^2+^ currents is set to -80 mV and 60 mV, respectively. In the case of VA5, VB6, VD5, we included in the models only the currents whose contribution to whole-cell dynamics has been characterized experimentally in [32].

Channel gene	Ion selectivity	AIY $\bar{g}$ [nS]	AVAL $\bar{g}$ [nS]	AVAR $\bar{g}$ [nS]	RIM $\bar{g}$ [nS]	VA5 $\bar{g}$ [nS]	VB6 $\bar{g}$ [nS]	VD5 $\bar{g}$ [nS]
*shl-1*	*K* ^*+*^	0.5	-	-	0.94	-	-	-
*shk-1*	*K* ^*+*^	-	-	-	-	0.1	0.4	1.2
*kvs-1*	*K* ^*+*^	-	-	-	-	-	-	-
*egl-2*	*K* ^*+*^	-	-	-	0.15	-	-	-
*egl-36*	*K* ^*+*^	-	-	-	-	-	-	-
*kqt-1*	*K* ^*+*^	0.2	-	-	-	-	-	-
*kqt-3*	*K* ^*+*^	-	-	-	-	-	-	-
*exp-2*	*K* ^*+*^	-	-	-	-	-	-	-
*irk-1/3*	*K* ^*+*^	-	0.1	0.04	0.34	1	1	0.7
*unc-103*	*K* ^*+*^	-	-	0.04	-	-	-	-
*slo-1*	*K* ^*+*^	1, 0.92	-	-	-	-	0.2	-
*slo-2*	*K* ^*+*^	-	-	-	-	3,3	1.75, 2	1.7,1.7
*kcnl-1/4*	*K* ^*+*^	-	-	-	-	-	-	-
*cca-1*	*Ca* ^*2+*^	-	-	-	0.87	-	-	0.1
*unc-2*	*Ca* ^*2+*^	-	-	-	0.33	-	0.1	-
*egl-19*	*Ca* ^*2+*^	0.1	0.10	0.064	0.1	0.15	0.1	0.9
*nca-1/2*	*Na* ^*+*^	0.06	0.03	0.05	-	0.01	0.03	0.09
${\bar{g}}_{leak}$ [nS]		0.14	0.15	0.23	0.1	0.1	0.13	0.2
*E*_*rev*_ [mV]		-89.57	-39.00	-37.00	-50.00	-70	-52	-75
*C*_*m*_ [pF]		1.05	9.66	8.43	1.55	5.84	7.87	3.52

It is worth to observe that, in most of the neurons, the final model includes a subset of the channels expressed in the neuron. As a first reason for it, we restricted the number of potassium currents by including one or a maximum of two currents per specific type (i.e., fast transient, non-inactivating, K-Ca, and irk) to facilitate the fitting procedure. Moreover, the fitting procedure predicts for certain currents a very small contribution, corresponding to one or less than one channel, so that their removal does not affect the whole-cell dynamics. In this last case, the conductance was set to zero, and therefore the current is removed from the model.

Once the optimal set of conductances is found, the behavior of each neuron is studied with current and voltage-clamp protocols. These protocols consist of multiple current or voltage steps, whose amplitude and duration match the experimental protocols. Moreover, we simulate the voltage- and current-clamp responses of *in silico* knockouts (KOs) for each neuron by suppressing the contribution of one current at a time. This study helps to elucidate the contribution of each current to the overall dynamics of the cell by mimicking the effect of pharmacological blockers. It is worth underlining that *in silico* knockout models cannot be directly compared to recordings on mutants for a certain gene, because mutant animals might rearrange the gene expression to overcome the misfunctioning of a single gene.

All the simulations are performed with NEURON in Python, and the results are analyzed in Python and MATLAB (2020Rb).

## Results

This section describes the models of the three interneurons, AVA, RIM, and AIY, and the models of the three motor neurons, VA5, VB6, and VD5. In particular, for each model, we simulate the responses of WT neurons to voltage- and current-clamp protocols specifically designed to reproduce the experimental data on which we fitted the models. Moreover, the role of each ionic current in the neuron dynamics is also studied by simulating the responses of KO neurons, in which we suppressed the contribution of one ionic current at a time.

### AVA interneurons

**Fig 1:**
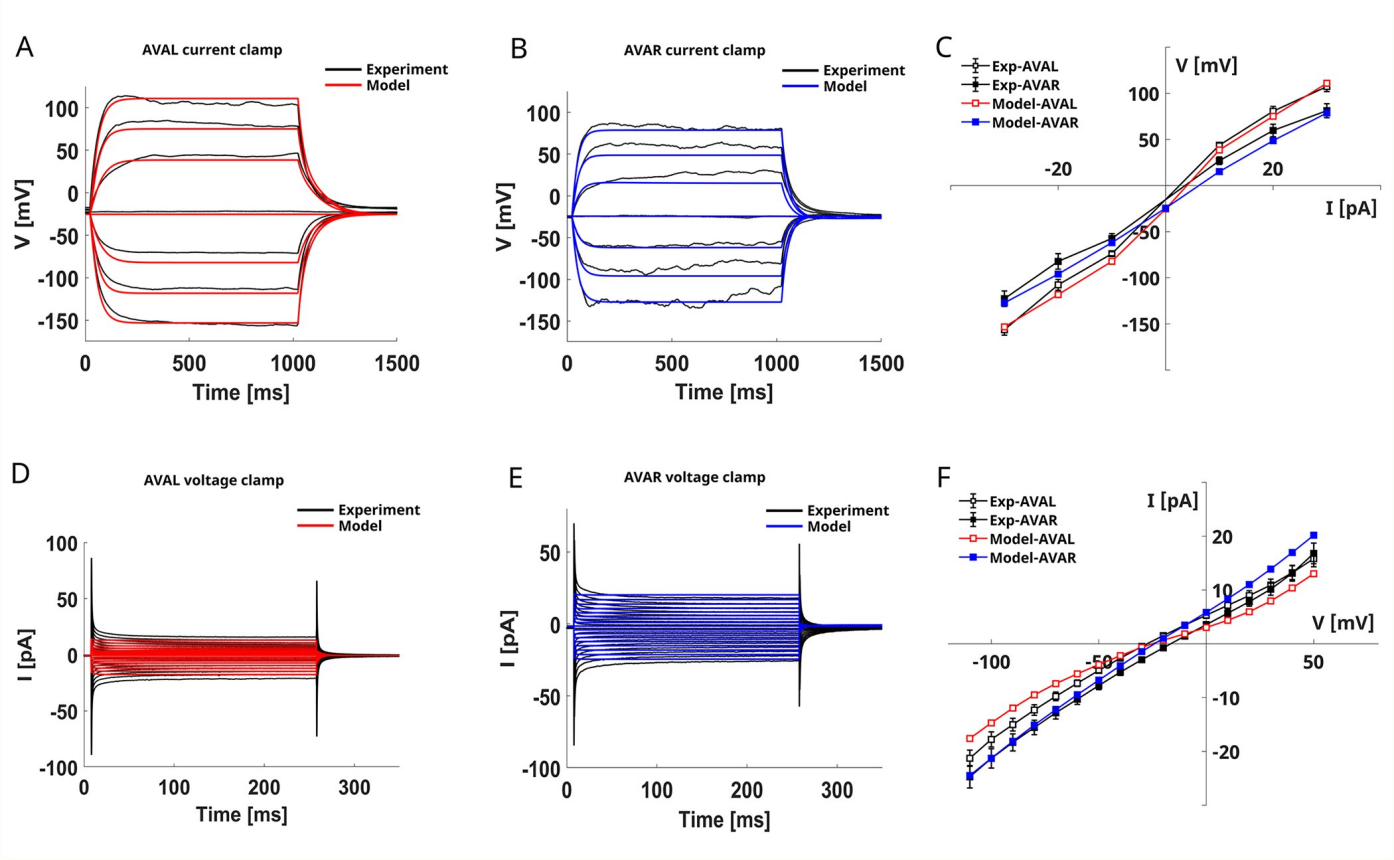
AVAL and AVAR models. **A) AVAL current-clamp simulation.** AVAL current clamp (red lines) simulation is compared to the corresponding mean experimental current clamp [29]. The simulation protocol is the same as the experimental recording, consisting of 7 current steps from -30 pA to 30 pA with a duration of 1000 ms (black lines). **B) AVAR current-clamp simulation.** AVAR simulated current-clamp responses (red lines) are compared to the corresponding mean experimental current-clamp (black lines) [29]. The simulation protocol, as the experimental one, consists of 7 voltage steps from -30 pA to 30 pA with a duration of 1000 ms. **C) AVAL and AVAR V-I curves**. Experimental curves from [29] are compared with simulated V-I curves computed from the simulated voltage responses shown in panels A and B. **D) AVAR voltage-clamp simulation.** AVAR voltage-clamp (red lines) simulation is compared to the corresponding experimental recordings from [29]. The simulations protocol replicates the experimental one consisting of 16 voltage steps ranging from -120 mV to 50 mV with a duration of 500 ms. **E) AVAR voltage-clamp simulation.** AVAR simulated whole-cell currents (red lines) are compared to the corresponding experimental currents (black lines) [29]. Same stimulation protocol of panel D). **F) AVAL and AVAR I-V curves**. Experimental steady-state I-V curves from [29] are compared with the simulated steady-state I-V curves computed from the simulated currents shown in panels D and E. The models were fitted on experimental current-clamp data obtained from [29], and shown in black in panels A and B.

AVA interneurons are a class of premotor interneurons critical for regulating backward locomotion [29]. Experimental whole-cell recording performed by Liu *et al*.[29] shows that AVAL and AVAR neurons have similar behavior both in voltage- and current clamp recordings (**Fig 1**, black lines) and have a depolarized resting potential. During current-clamp experiments, AVAL and AVAR neurons display symmetric responses to hyperpolarizing and depolarizing stimuli, resembling those of a passive RC-circuit, as also confirmed by the near-linear V-I curve. Despite the similarities between the two neurons, current injections elicit larger voltage excursions in AVAL than in AVAR (**Fig 1A–1C**, black lines). The linear behavior of the neurons is also reflected in the voltage-clamp recordings, that show linear whole-cell currents in both neurons with slightly larger currents in AVAR than AVAL (**Fig 1D–1F**, black lines).

**Fig 1** compares the AVAL and AVAR neuron models with the corresponding experimental data from [29]. Overall, our models, even with only five currents (NCA, EGL19, IRK, UNC103, and LEAK), reproduce the experimental current clamp recordings (**Fig 1A and 1B,** red and blue lines), as also demonstrated by the V-I curves (**Fig 1C,** red and blue lines). Both AVAR and AVAL exhibit a near-linear input-output relation (**Fig 1C**) so that hyperpolarizing and depolarizing stimuli of the same magnitude produce similar voltage excursions, with AVAL more sensitive than AVAR to both kinds of stimuli (**Fig 1A and 1B,** red and blue lines). The current-clamp responses of both neurons are characterized by a slow-rising phase (~200 ms) followed by a stable plateau that is sustained until the stimulus is removed. The repolarization of the neuron is smooth, with a time scale comparable to that of the rising phase. We also simulate the voltage-clamp recording (**Fig 1D–1F,** red and blue lines). As expected, the linear behavior observed in the current clamp recording is also conserved in the voltage-clamp, as shown by I-V relations (**Fig 1E,** red and blue lines). Overall, despite they have been optimized to reproduce the current-clamp recordings, our models reproduce properly the features of the whole cell currents (**Fig 1**D and 1E). The main differences with the experimental data are observed in AVAL, where the simulated currents are sightly overestimated for hyperpolarizing stimuli and underestimated for depolarizing stimuli (**Fig 1**D and 1F).

**Fig 2:**
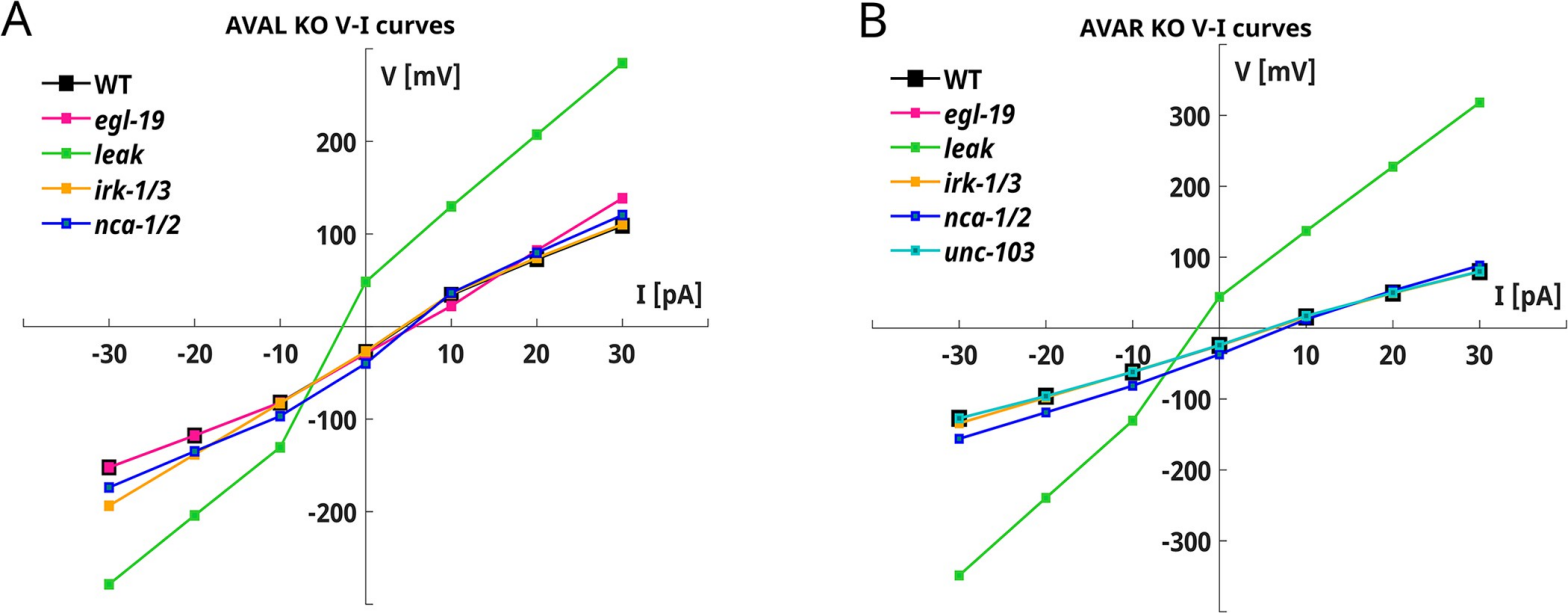
AVAL and AVAR KO simulations. **A) AVAL KO neurons V-I curves.** The V-I curves of KO neurons are computed from the KO current-clamp simulations shown in **S1 Fig**. **B) AVAR KO neurons V-I curves.** The V-I curves of KO neurons are computed from the KO current-clamp simulations shown in **S2 Fig**.

Next, we analyze the responses of KO neurons to provide insights into the role of specific ionic currents in shaping the responses of AVAL and AVAR interneurons (**Figs 1 and 2**, **S1** and **S2 Figs**).

The leakage current (in green) is critical for defining the resting potential and for the overall neuron dynamics (**Fig 2**). Indeed, its suppression shifts the resting potential to ~50 mV and strongly influences the repolarization phase for hyperpolarizing stimuli (**S1B and S2B Figs**). In AVAL neurons, the EGL-19 currents (in magenta) are important for defining the plateau state for depolarizing stimuli, while IRK-1/3 currents (in orange) mainly influence the shape of the responses to hyperpolarizing stimuli (**S1A and S1C Fig**). In contrast, in AVAR neurons, EGL-19 (in magenta) suppression does not cause significant changes in the responses (**Fig 2A, 2B** and **S2A Fig**), whereas the role of IRK currents (in orange) is conserved even though their effect is less strong compared to that observed in AVAL neurons (**Fig 2A, 2B** and **S2C Fig**). Finally, the suppression of NCA currents (in blue) has similar effects in both neurons, causing a downward shift in the resting potential (~-40 mV in AVAL and ~-45 mV in AVAR) but not altering the shape of the responses (**Fig 2**, **S1D** and **S2D Figs**).

### AIY interneurons

**Fig 3:**
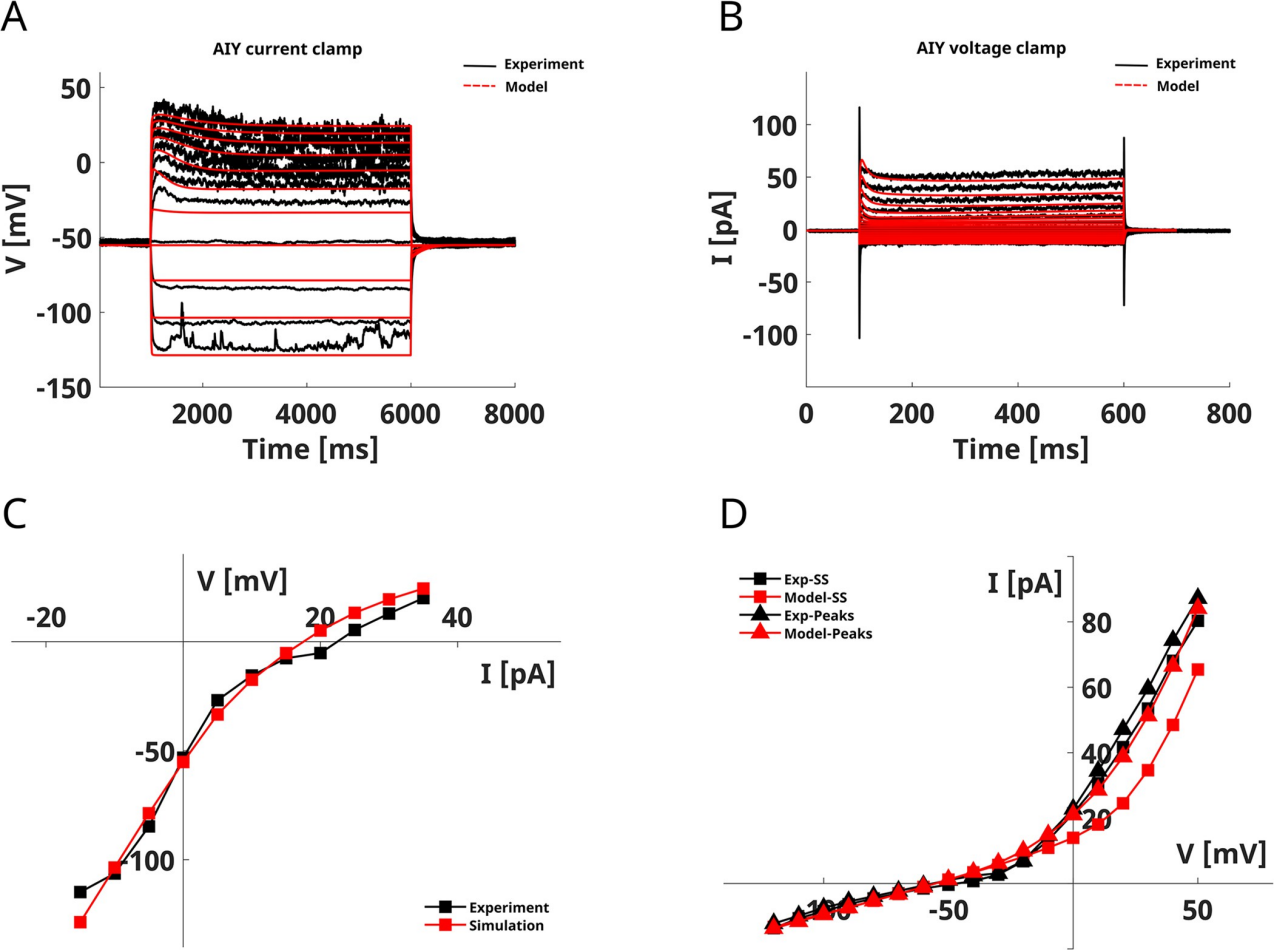
AIY model. **A) AIY current-clamp simulation.** The panel shows the comparison of the experimental recording (in black) on AIY neurons [30] and the simulated current-clamp responses (in red). The simulation protocol reproduces the experimental one consisting of 11 current steps ranging from -15 pA to 35 pA with a duration of 5000 ms. **B) AIY voltage-clamp simulation.** The figure shows the average experimental (in black, from [30]) and the simulated (in red) whole-cell currents in AIY neurons. The voltage clamp protocol consisted of 16 voltage steps from -120 mV to 50 mV with a duration of 500 ms. **C), D) AIY V-I and I-V curves**. The V-I and steady-state (SS) I-V curves are computed by averaging the voltage and the current in the last 10 ms of the stimulation step, respectively. The peaks I-V curve is computed by finding the maximal current in the first 50 ms of stimulation. The red line and squares represent the model output, while the experimental data (from [30]) are represented in black. The model was fitted on experimental current-clamp data obtained from [30] and shown in black in panel A.

AIY interneurons are amphid interneurons postsynaptic to many olfactory and gustatory neurons of the head. Electrophysiological recordings by Liu *et al*. [30] showed that AIY neurons do not originate regenerative responses, rather they have an enhanced responsiveness to hyperpolarizing stimuli (**Fig 3A and 3B**, black lines). In voltage-clamp configuration, AIY neurons display membrane currents dominated by an outward rectifier component with a small contribution of inward rectifier currents (**Fig 3**A and 3B, black lines).

We model AIY neurons with six ionic currents, including three potassium currents, SHL1, KQT1, and SLO1, one voltage-gated calcium current, EGL19, one sodium current NCA, and the LEAKAGE current.

The model correctly reproduces the voltage response of the neuron for both hyperpolarizing and depolarizing stimuli (**Fig 3**A, red lines). As in the experiments, the neuron is more sensitive to hyperpolarizing than to depolarizing stimuli, and for depolarizing stimuli, it shows a slow upstroke followed by a plateau. Despite the non-linearity of the V-I curve (**Fig 3**C), the neuron does not display threshold regenerative responses but rather a rectifying behavior at high stimulus intensities.

Voltage-clamp simulations, show that the model also reproduces the outward rectifying behavior of the average whole-cell currents (**Fig 3**B and 3D, red lines), but with a slight underestimation of the steady-state current.

**Fig 4:**
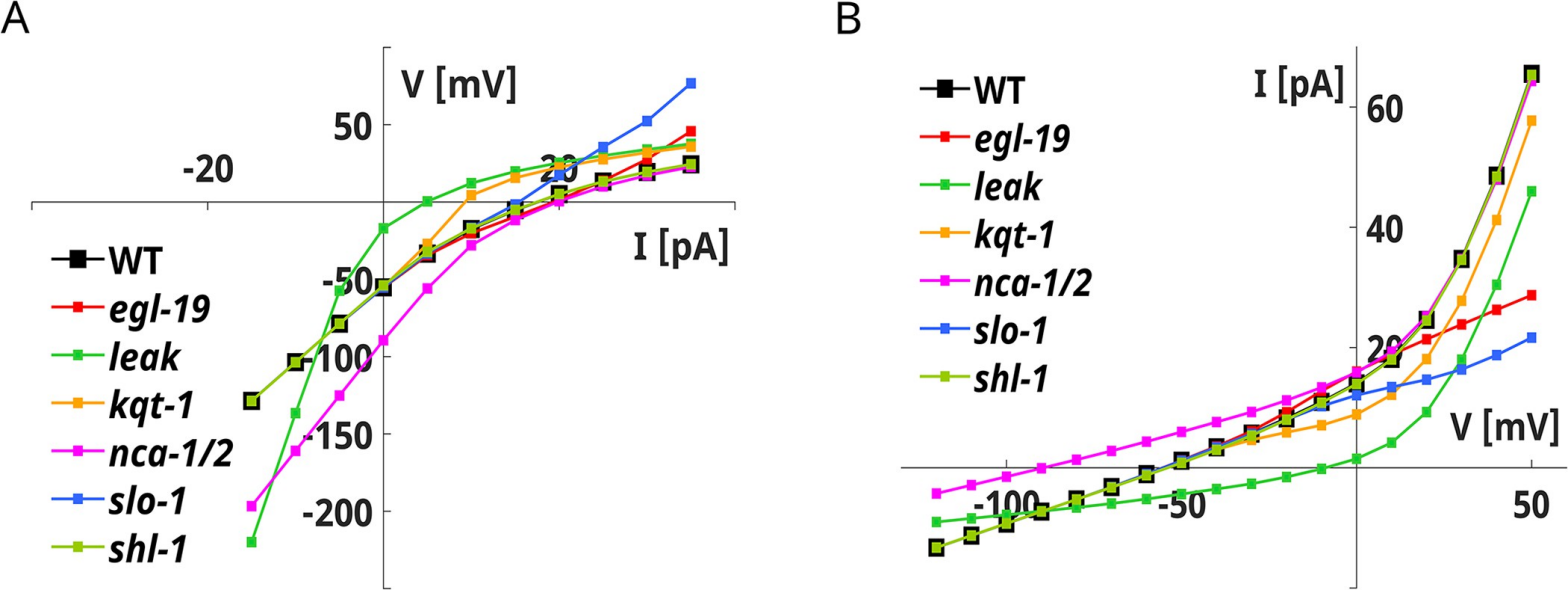
AIY KO neurons V-I and I-V curves. **A) AIY KO neurons V-I curves.** The V-I curves of KO neurons are computed from the current clamp simulations shown in **S3 Fig**. **B) AIY KO neurons I-V curves.** The I-V curves of KO neurons are computed from the voltage clamp simulations shown in **S4 Fig**.

We studied KO neurons to elucidate the origin of these responses both in the current- and voltage-clamp configuration (**Fig 4**, **S3 and S4 Figs**).

Our simulations indicate that SLO1 currents are essential for AIY behavior. Their removal significantly suppresses the steady-state currents (**Fig 4**B, **S4**C **Fig**). In voltage-clamp simulations, the SLO1 removal significantly alters the responses to depolarizing stimuli, indicating that they prevent abnormal membrane potential growth (**S4**C **Fig**). KQT1 currents, with SHL1 currents, play a secondary role in defining the steady-state and the fast transient currents (**S4**A and S4B **Fig**). SHL1 removal mainly alters the upstroke phase of the responses, while KQT1 removal sightly increases the plateau level (**S3**A and S3B **Fig**). EGL-19 calcium currents are recruited in the upstroke phase of the membrane potential and are essential for the proper functioning of SLO1 currents (**S3**E and **S4**E **Figs**). Finally, leakage and NCA currents are critical for resting potential definition and for ensuring the proper functioning of the neuron to depolarizing stimuli (**S3**D, S3F **Fig** and **S4**D, S4F **Fig**).

### RIM interneurons

**Fig 5:**
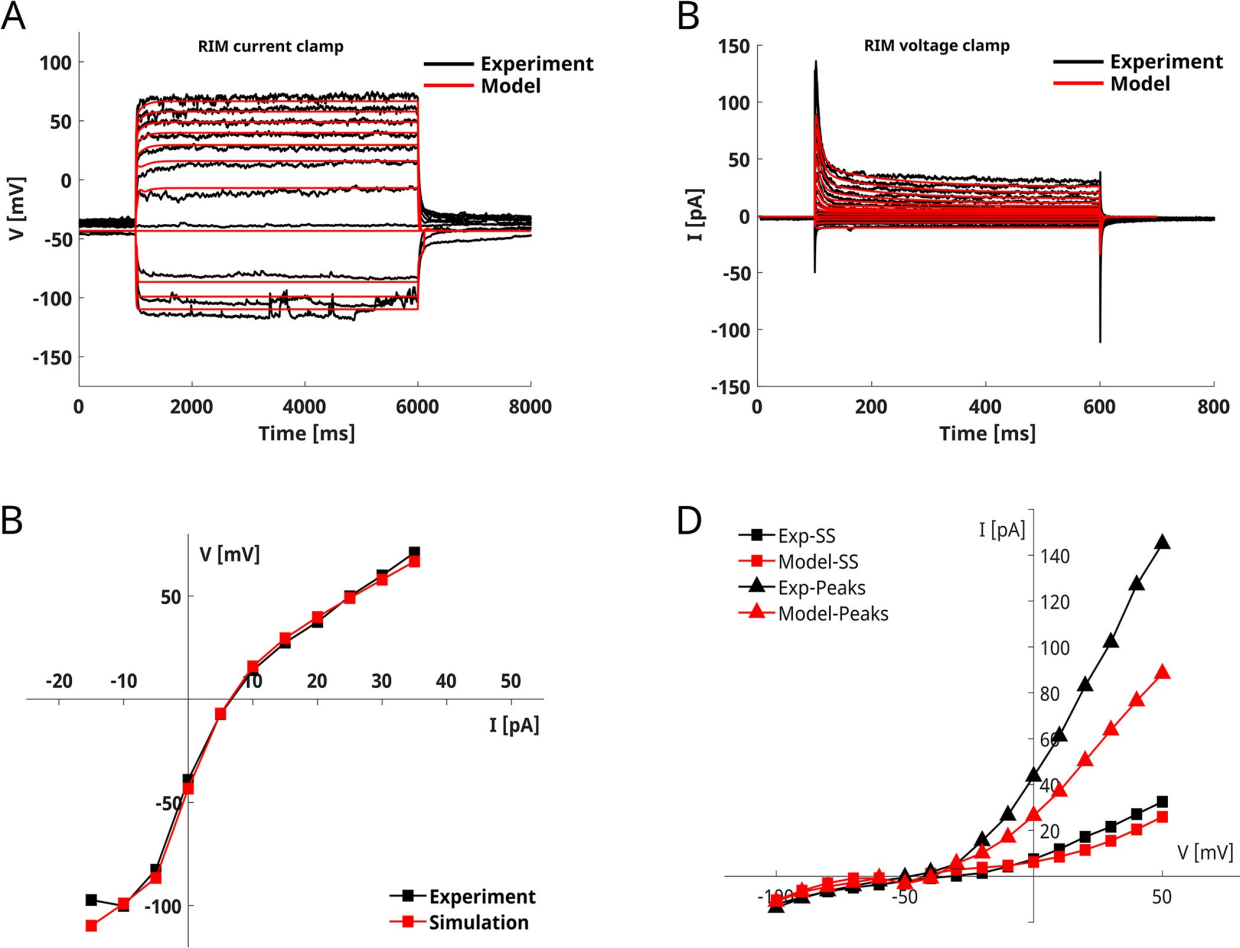
RIM model. **A) RIM current-clamp simulation.** The panel shows the comparison of the experimental recording (in black) on RIM neuron [30] and the simulated current clamp responses (in red). The simulation protocol reproduces the experimental one consisting of 11 current steps ranging from -15 pA to 35 pA with a duration of 5000 ms. **B) RIM voltage-clamp simulation.** The figure shows the experimental (in black, from [30]) and the simulated (in red) whole-cell currents in RIM neurons. The voltage-clamp protocol consisted of 16 voltage steps from -100 mV to 50 mV with a duration of 500 ms. **C), D) RIM V-I and I-V curves**. The V-I and steady-state (SS) I-V curves are computed by averaging the voltage and the current in the last 10 ms of the stimulation step, respectively. The peaks I-V curve is computed by finding the maximal current in the first 50 ms of stimulation. The model was fitted on experimental current- and voltage-clamp data obtained from [30] and shown in black in panels A and B.

RIM neurons are a class of interneurons involved in locomotion regulation. In current-clamp recordings, [30] RIM neurons show smooth responses to both depolarizing and hyperpolarizing stimuli, with marked sensitivity to hyperpolarizing stimuli as AIY neurons (**Fig 5**A, black lines). The whole-cell currents display a fast-activating component that rapidly degrades in a non-inactivating component. For hyperpolarizing stimuli, the behavior is characterized by small inward currents (**Fig 5**B, black lines). For these peculiar characteristics of the whole-cell currents RIM neurons are classified as “transient outward rectifying” neurons [62].

We model the RIM neuron with a set of seven currents, including the three calcium currents EGL19, UNC2, and CCA1, the transient potassium current SHL1, the non-inactivating potassium current EGL-2, the inward rectifier current IRK, and the leakage current. The model correctly reproduces RIM responses upon current injections from -15 to 35 pA (**Fig 5A**). As in the experimental recordings, the neuron is more sensitive to hyperpolarizing than depolarizing stimuli (**Fig 5A**). For both hyperpolarizing and depolarizing stimuli, the voltage rises smoothly and stabilizes to the steady-state value until the stimulus is removed (**Fig 5A**).

**Fig 6:**
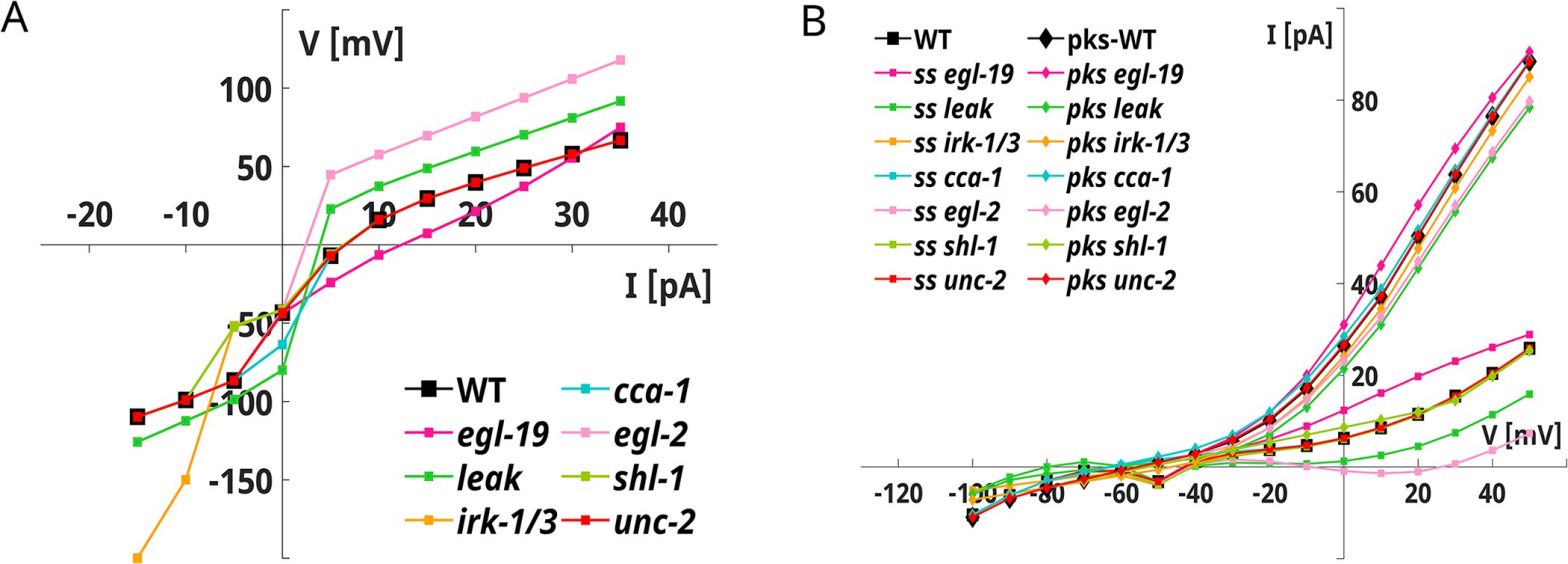
RIM knock-out simulations. **A) RIM KO neurons V-I curves.** The V-I curves of KO neurons are computed from the current-clamp simulations shown in **S6 Fig**. **B) RIM KO neurons I-V curves.** The I-V curves of KO neurons are computed from whole-cell currents shown in **S5 Fig**.

Our model is also consistent with the experimental voltage-clamp recordings and reproduces both fast transient and steady-state non-inactivating currents (**Fig 5B and 5D**). To dissect the role of each ionic current in RIM dynamics, we analyze the responses of KO neurons in the current and voltage-clamp configuration (**Fig 6, S5** and **S6 Figs**). Our results indicate that EGL-2 currents (in pink) are responsible for the steady-state non-inactivating currents observed in the voltage-clamp (**Fig 6** and **S5B Fig**). As expected, the SHL-1 (in green) and IRK currents (in orange) drive the fast transient and inward components, respectively (**Fig 6, S5A and S5C Fig**).

These currents also influence the responses to hyperpolarizing stimuli, as shown in **S6 Fig,** while EGL2 modulates the resting potential and the responses to depolarizing stimuli (**Fig 6** and **S6B Fig**). Concerning the calcium currents, the suppression of UNC2 currents (in red) does not significantly alter the current and voltage-clamp responses. In contrast, EGL19 (in magenta) removal shifts upward the steady-state voltage for depolarizing stimuli (**Fig 6** and **S6F Fig**) and increases the outward currents (**F**). CCA1 calcium channels (in cyan) mostly influence the resting potential, shifting it downward to ~ -64 mV (**Fig 6A and S6E Fig**). Finally, the LEAK current (in dark green) shifts the resting potential to -80 mV and induces bistability in the current-clamp responses **(Fig 6** and **S6D Fig**).

### VA5-VB6-VD5 motor neurons

**Fig 7:**
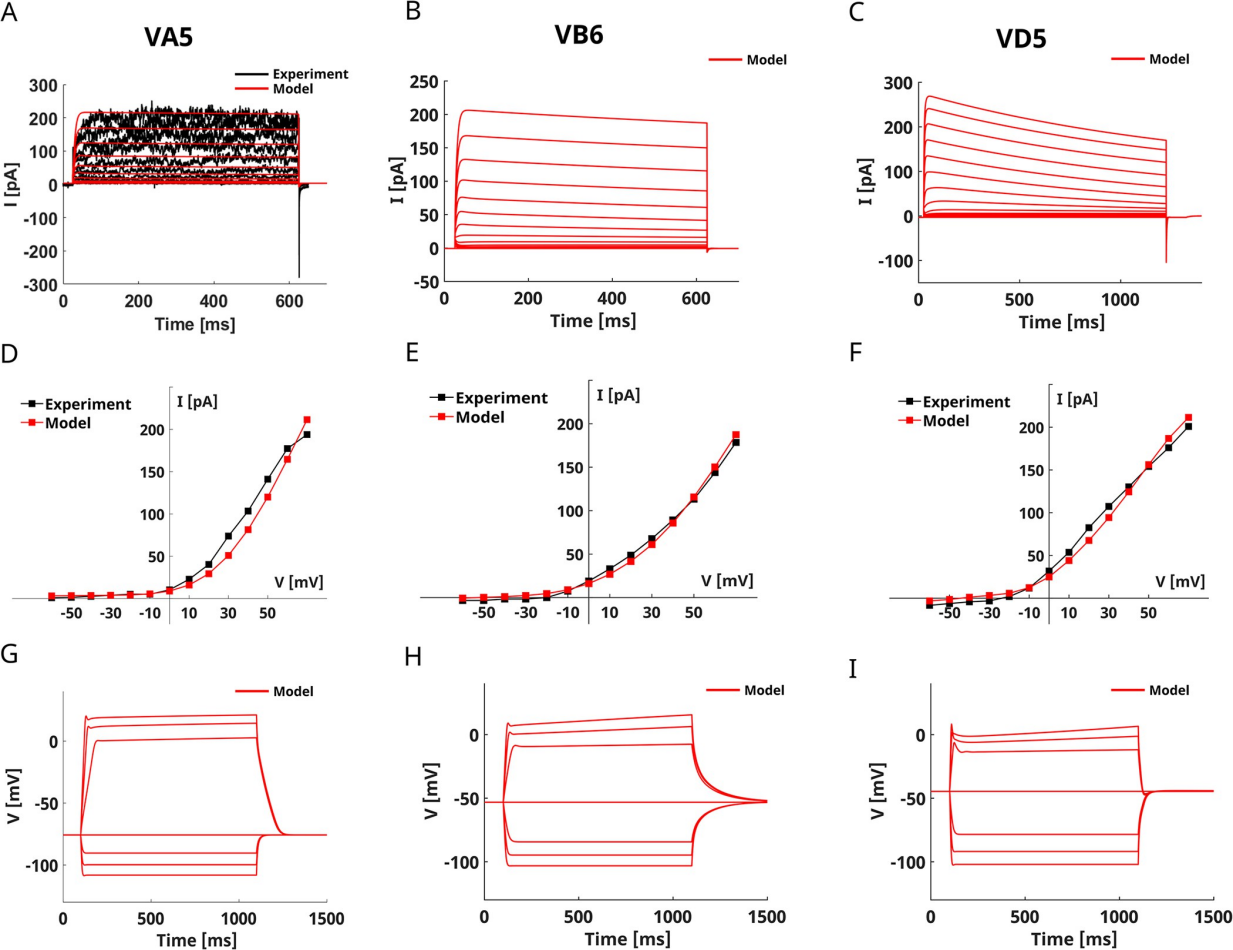
VA5, VB6, and VD5 models. **A) VA5 voltage-clamp simulation.** The panel compares the experimental whole-cell currents (in black) of VA5 neurons [40] with the corresponding simulated currents shown in red. **B) VB6 voltage-clamp simulation.** The panel shows VB6 currents obtained in a voltage-clamp simulation. **C) VD5 voltage-clamp simulation.** The panel shows VD5 currents obtained in a voltage-clamp simulation. All the voltage-clamp simulations shown in panels A, B, and C are performed using the same protocol that replicates the experimental one consisting of 14 voltage steps ranging from -60 mV to 70 mV with a duration of 5000 ms. **D) VA5 Steady-State I-V curves.** The simulated steady-state I-V curve (in red) is compared to the corresponding experimental curve (in black) from [40]. **E) VB6 Steady-State I-V curve.** The simulated (in red) steady-state I-V curve of VB6 is compared to the corresponding experimental curve (in black) from [32]. **F) VD5 Steady-State I-V curve.** The simulated (in red) steady-state I-V curve of VD5 is compared to the corresponding experimental curve (in black) from [32, 40]. **G) VA5 current-clamp simulation.** The panel shows the VA5 voltage responses to current steps ranging from -30 pA to 30 pA with 10 pA increments. The simulation protocol has been selected to match the electrophysiological recordings shown in [39, 29]. **H) VB6 current-clamp simulation**. The panel shows the predicted VB6 voltage responses to current steps ranging from -30 pA to 30 pA with 10 pA increments. **I) VD5 current-clamp simulation**. The panel shows the predicted VD5 voltage responses to current steps ranging from -30 pA to 30 pA with 10 pA increments. The model of VA5 was fitted on the voltage-clamp data obtained from [40] and shown in panel A. Instead, the models of VB6 and VD5 were fitted on the I-V curves obtained from [32].

VA5, VB6, and VD5 are representative of A-, B-, and D-class motor neurons. Voltage-clamp recordings by Yuan *et al*. show that these neurons are characterized by outward rectifier currents mainly driven by SLO-2 channels. The whole-cell currents of the three neurons display slow activation followed by a small and slow inactivation [32, 40], and a very small inward component (**Fig 7**). To the best of our knowledge, no current-clamp recordings have been published for VB6 and VD5 neurons, while for VA5 neurons both spontaneous activity recordings and current-clamp recordings suggest a bistable behavior [32, 39]. VA5 neurons display a reduced sensitivity to hyperpolarizing stimuli, while they show threshold responses to depolarizing stimuli with a fast increase and a slow repolarization [39]. Interestingly, in VA5 neurons the recovery from hyperpolarizing stimuli is faster than to depolarizing stimuli [39].

We model the three neurons with the same set of ionic currents, including isolated and coupled SLO2 and SLO1 K-Ca currents, SHK1 and IRK potassium currents, voltage-gated calcium currents (EGL19, UNC2, CCA1), NCA, and leakage currents. The VA5, VB6, and VD5 models capture the main features of the experimental recordings [32], showing large outward rectifiers and small inward currents (**Fig 7A–7G**). In addition, VD5 shows a fast transient component that is not observed in VA5 and VB6. We also simulate the responses of the three neurons upon current injections from -30 pA to 30 pA (**Fig 7G–7I**). The VA5 responses agree with experimental recordings from [39]. The neuron shows smooth hyperpolarizing and depolarizing responses and presents a pronounced sensitivity to depolarizing stimuli (**Fig 7G**). Indeed, the membrane potential excursion for a 30 pA stimulus is around 100 mV, compared to an excursion of 30 mV for the -30 pA stimulus. In accordance with experimental data [39], VA5 neurons repolarize faster after hyperpolarizing stimuli than depolarizing stimuli (**Fig 7G**). Current-clamp simulations performed on VB6 predict smooth responses to hyperpolarizing and depolarizing stimuli (**Fig 7H**). The neuron is more sensitive to depolarizing than hyperpolarizing stimuli, and its resting potential (-53.2 mV) is in agreement with experimental data. Compared to VA5, VB6 neurons repolarize slowly after hyperpolarizing stimuli, and the repolarization time is similar for hyperpolarizing and depolarizing stimuli (**Fig 7H**). The predicted current-clamp responses for VD5 show smooth hyperpolarization with voltage excursions similar to VB6 (**Fig 7I**). In contrast to VA5 and VB6, the responses to depolarizing stimuli show an initial peak followed by a slow increase of the membrane voltage (**Fig 7I**). Finally, compared to VA5 (-75.2 mV) and VB6 (-53.19 mV), VD5 neurons have a depolarized resting potential of around -44.61 mV, in accordance with experimental data [32].

**Fig 8:**
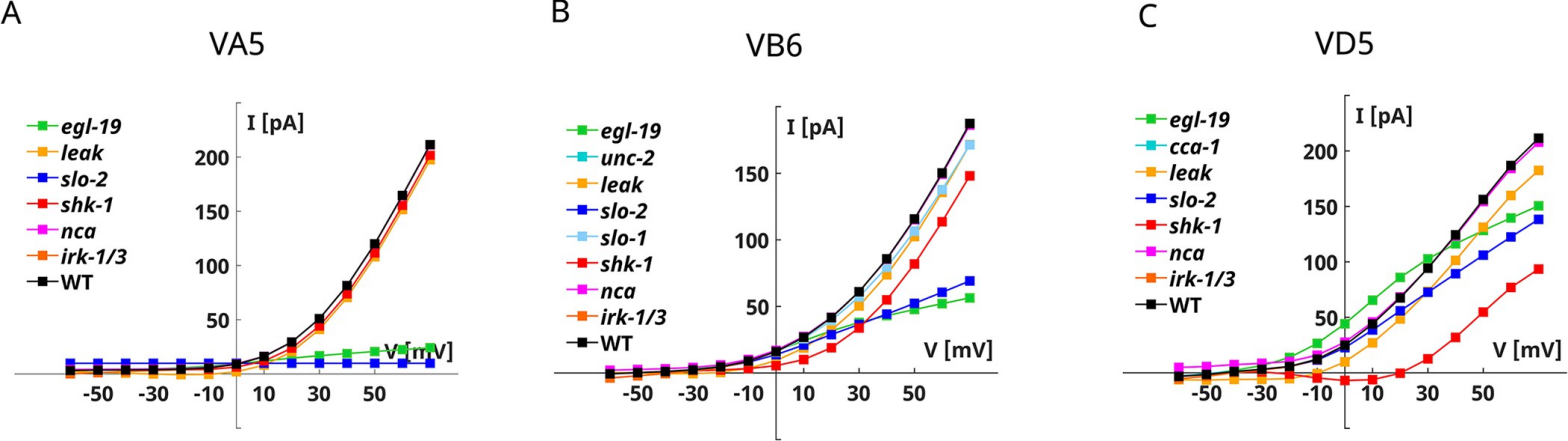
VA5, VB6, and VD5 knockout I-V curves. **A) VA5 KO neurons I-V curves**. The panel shows the steady-state I-V curves obtained with voltage clamp simulations on KO neurons shown in **A-F**. **B) VB6 KO neurons I-V curves**. The panel shows the steady-state I-V curves obtained with voltage clamp simulations on KO neurons shown in **S8A–S8J Fig. C) VD5 KO neurons I-V curves**. The panel shows the steady-state I-V curves obtained with voltage clamp simulations on KO neurons shown in **S9A–S9H Fig**. The simulation protocol is the same for the three neurons and consists of 14 voltage steps ranging from -60 mV to 70 mV with a duration of 5000 ms.

For these three motor neurons, a complete electrophysiological characterization has been performed by Liu *et al*. with voltage clamp experiments [32]. Therefore, we replicate this analysis with KOs simulations in the voltage-clamp configuration to test the quality of our models. Furthermore, we study the role of the different ionic currents in the predicted voltage responses by simulating the current-clamp responses of KO neurons. Voltage-clamp simulations of *in silico* KO neurons confirmed that the principal contribution to the outward currents in VA5 and VB6 neurons is given by SLO-2 currents (**Fig 8As, 8B, S7A–S7F Fig,** and **S8A–S8J Fig,** in blue) [32]. Also, in the case of VD5 neurons, our model correctly portrays the dominant role of SHK1 (in red) instead of SLO2 currents in shaping the whole-cell currents **(Fig 8C** and **S9A–S9H Fig)** [32]. Moreover, the three models also highlight the importance of EGL19 calcium currents (in green) in ensuring the proper functioning of SLO2 channels (**Fig 8**, **S7–S9 Figs.**) [32].

Next, we analyze the role of the ionic currents in the current-clamp responses (**S7–S9 Figs**). Firstly, we note that the leakage and NCA currents have a minor influence on the simulated currents, but they are critical in defining the resting potential and preventing intrinsic bistability of the neurons **(S7I, S7J Fig, S8L, S8P Fig,** and **S9H, S9M Fig,** in magenta**).** As expected from the voltage-clamp simulations, SHK1 currents (in red) are critical for VD5 responses entailing bistability in the resting potential of the neuron. In contrast, SHK1 currents have a minor influence on VA5 and VB6 responses, influencing the plateau level and the repolarization time (**S7K and S8O Figs**). EGL19 calcium currents (in green) are involved in the responses to depolarizing stimuli, particularly as far as VA5 neurons are concerned (**S7G, S8H and S9I Figs**). Despite their essential contribution to the outward currents in the voltage-clamp recordings, SLO2 (in blue) suppression has significant effects only on VA5 voltage dynamics, while VB6 and VD5 are less affected by their removal (**S7H, S8M and S9K Figs**). Notwithstanding their small conductance, IRK currents (in orange) are critical for shaping the responses to hyperpolarizing stimuli in the three neurons preventing abnormal hyperpolarization (**S7L, S8P and S9N Figs**).

## Discussion and conclusion

In this section, we discuss the results presented in the previous section and compare them with available literature and existing models of *C*. *elegans* neurons. The biophysical models here proposed were overall able to reproduce main features of electrophysiological data. For each neuron, we select the set of ionic currents based on gene expression data, and we obtain a set of conductances by fitting experimental whole-cell data from literature [29, 30, 32, 40]. When possible, we prefer to fit the models on current-clamp data because we are interested in the study of the voltage responses of the neurons upon current injection. Therefore, we select the one that successfully reproduces the current-clamp responses among the different sets of parameters that could be obtained in the optimization procedure.

The AVAL/AVAR models reproduce the voltage responses of the neurons to current injections (**Fig 1**A–1C). The behavior of the two neurons in current-clamp experiments resembles that of a passive RC circuit, mainly defined by passive leakage currents. Despite slight differences, the near-linear behavior of the neuron is also reflected by voltage-clamp simulations (**Fig 1**D–1F).

Simulations on KO neurons highlighted a dominant role of leakage currents in defining the resting potential of AVAL and AVAR neurons **(S1B** and **S2B Figs).** Indeed, for both neurons the resting potential, in the absence of leakage currents, is considerably depolarized. This shift in the resting potential might be related to a limitation of our mathematical description of the neuron, which does not include any other potassium current that could compensate for the loss of the leakage current. Therefore, the resting potential is defined mainly by the calcium reversal potential, that is 60 mV, with a small contribution of the IRK potassium currents. Moreover, it has to be taken into account that, in living worms, this strong depolarization might not be observed, due to compensation mechanisms that prevent abnormal shifts of reversal potential. This aspect also deserves further investigation considering recent results suggesting that the resting potential is influenced by the voltage-insensitive K2P channel TWK-40 [50]. The AVAL resting potential (-25.4 mV) is in agreement with the experimental data [29]. In this context, our model correctly reproduces this phenomenon by indirectly including in the leakage term this passive contribution to ionic current. In the case of AVAR, our model agrees with the mean resting potential obtained in the current-clamp recordings [29, 31, 50] but not with the mean value recorded in voltage-clamp [29]. Furthermore, our models suggest that some physiological differences might exist between AVAL and AVAR neurons, despite their similarities in the responses and in the set of ionic currents used in models. Indeed, as shown by the KO current-clamp simulations (**S1B** and **S2B Figs**), AVAL responses are more influenced by voltage-gated currents (EGL19 and IRK) than AVAR responses which are, instead, almost entirely shaped by voltage-insensitive currents (NCA and LEAK). Overall, the I-V curves of AVAL and AVAR display a linear behavior (**Fig 1**). Taken together with computational studies [75], this result might suggest that the spontaneous bimodal distribution of the AVA voltage observed experimentally [76] is more likely related to a bistable synaptic input than to the physiological properties of the neurons.

The AIY model reproduces the voltage responses of the neuron, in particular to hyperpolarizing stimuli. The model also reproduces the slow responses to depolarizing stimuli and the average voltage-clamp responses, but with a slight underestimation of the steady-state currents (**Fig 3**) [30]. The analysis of the KO neurons suggests that the responses to depolarizing stimuli are mainly influenced by EGL19 and SLO1 currents with a small contribution of SHL-1 currents in the initial phase (**S3 Fig**). Despite the absence of IRK currents, the model reproduces the enhanced sensitivity to hyperpolarizing stimuli, a peculiar feature of AIY neurons. In accordance with the already published model of AIY [34] our model includes the contribution of a persistent (KQT-1) potassium current that influences the responses to depolarizing stimuli (**S3 Fig**). However, with respect to [34] we also include the fast potassium current SHL1 and calcium-activated potassium current SLO1. The SLO1 current, in particular, is critical for defining the outward rectifying behavior of the neuron (**S3** and **S4 Figs**). Concerning the calcium currents, our model matches the AIY experimental data using slowly activating (EGL19) calcium currents in accordance with available gene expression data for AIY in the Wormbase and CENGen databases [67, 68]. In contrast, the model of AIY by Naudin *et al*. includes the contribution of a transient calcium current that might be identified with UNC2 or CCA1 currents. Our results suggest that the L-type persistent calcium current, not transient calcium currents, is relevant for AIY dynamics, in particular as far as the responses to depolarizing stimuli are concerned (**S3E Fig**).

Among the modeled neurons, RIM is reproduced with the highest accuracy both in voltage- and current-clamp configuration (**Fig 5**) [30]. Despite the discrepancy in the intensity of the peak currents observed in the voltage-clamp, the currents are overall in agreement with the experimental ones, showing a fast transient component that might be driven by SHL1 currents and small non-inactivating outward and inward components related to EGL2 and IRK, respectively (**S5A–S5C Fig**). Compared to already published models of RIM neurons, our model includes, in addition to transient calcium currents driven by UNC-2 and CCA-1 channels, a persistent calcium current from EGL-19 channels and predicts an important role of CCA1 currents in regulating the resting potential of the neuron. In contrast, UNC2 currents do not significantly contribute to neuron dynamics. Our model correctly reproduces the steady-state near-linear behavior of the neuron. This result is in accordance with a computational analysis showing that the RIM ON-OFF behavior observed upon odor stimulation is related to the synaptic input rather than to the intrinsic physiological properties of the neuron [13, 75]. In addition, our model suggests that the EGL-2 might be critical for preventing intrinsic bistability in the RIM dynamics (**S5B Fig**).

Finally, we also model the responses of three motor neurons, VA5, VB6, and VD5, sharing similar electrophysiological properties. The models adequately reproduce the voltage-clamp recordings (**Fig 7**), as confirmed by the analysis of KOs responses in the voltage-clamp configuration (**Fig 8**, **S7–S9 Figs**). The outward rectifier behavior of the three neurons is dominated by K-Ca currents driven by SLO-2 channels coupled with L-type (EGL-19) calcium currents and, in the case of VD5 neurons, by SHK1 voltage-gated potassium currents [32] (**Fig 8**, **S7–S9 Figs**). We also simulate the responses of the three neurons to current-clamp responses (**Fig 7**). In the case of VA5 neurons, our model correctly captures the features of the responses recorded in current-clamp experiments [39] and explains the role of the different ionic currents in the voltage responses. As expected, SLO2 and EGL19 currents suppression strongly influences the responses, together with LEAK and IRK currents (**S7G–S7L Fig)**. We also predict the current-clamp responses for VB6 and VA5 neurons and analyze their origin. The two neurons display smooth depolarizing and hyperpolarizing responses and are more sensitive to depolarizing than hyperpolarizing stimuli (**Fig 7H and 7I**). SHK1 and IRK currents influence the depolarizing and the hyperpolarizing responses, respectively, with minor contributions of EGL19 and SLO2 currents (**S8** and **S9 Figs**). As for RIM and AVA neurons, the recordings of spontaneous activity on VA5 show a bistable ON-OFF behavior, this behavior is consistent with spontaneous activations of the neuron that switches between the resting and plateau, excited, state depending on the synaptic input from the surrounding network [62]. Therefore, also in the case of VA5 neurons, the bistability in the spontaneous activity might be related to the specific synaptic input.

Summarizing, we have modelled the behavior of six nematode neurons in the single-compartment approximation. Our models capture the main features of the neurons both in the voltage- and current-clamp configuration. Despite their capabilities to describe the behavior of the neurons and the interplay of currents underlying the whole-cell behavior, it is important to discuss the limitations of this approach.

The first limitation of our study is related to the possible non-uniqueness of the set of parameters obtained with the optimization protocol. It is possible that multiple sets of parameters could reproduce the behavior of the neurons equally well. This degeneracy reflects the complexity of the mathematical models and of the biological systems. From the mathematical point of view, the best set of parameters is a subset of points in the space of parameters, and different subsets of points might represent the neuron with the same accuracy, reflecting the variability observed in the ionic channel expression. Indeed, from the biological point of view, neurons belonging to the same cell type show variability in the ion channel densities and express ionic channels with overlapping kinetic properties [77]. Despite the differences in the physiological properties, neurons of the same class could originate reliable and similar responses. This redundancy in the ion channels’ voltage and time characteristics may confer resilience to deletion, mutations, and pharmacological blocking [77]. Clearly, this complexity could not be reflected by a single set of conductances but rather by a distribution of parameter sets. This is still an open problem requiring new strategies and algorithms to explore a wider parameter space. A recent work by Gonçalves *et al*. applies machine learning and tools to fit the Hodgkin-Huxley model to electrophysiological data [78], opening interesting possibilities for future advances in the field of *C*. *elegans* neurons modeling.

As a second possible limitation, despite the fact that single-compartment models have been proven to be reliable in the case of *C*. *elegans* [30, 33, 34, 38], it has to be underlined that a complete description of the neurons functioning should include multi-compartments representing the different functionalities of the different regions of the cell (i.e. axon, soma and dendrites) [79]. However, to the best of our knowledge, no information is available on the specific distribution of the ionic channels in these neurons. Moreover, until today, few studies have focused on dissecting the different functionalities of the different biological regions of *C*. *elegans* neurons [54, 70, 71]. However, these studies are not specifically focused on the neurons modelled in this work and do not report information on the distribution of ionic channels in the compartments. Considering these two limiting aspects that are fundamental for the development of accurate multi-compartmental models, we decided to develop our model in the single-compartment approximation, which has been successfully applied for other nematode neurons.

As a third possible limitation, that is intrinsic to the single neuron model, the electrophysiology data are measured *in-vivo* and therefore include the effect of the surrounding network of cells and neurons. In the single neuron model, all these effects are treated as due to the neuron itself, somehow embedded and averaged. This is however not a great limitation, as far as all neurons in a network model are treated on the same foot, and if it is possible to identify and to disentangle the different molecular pathways and to separately model them, as is the case for the calcium dynamics.

In conclusion, to the best of our knowledge, the six biophysical models of interneurons here presented are the first explicitly including specific ion currents. Our models are in agreement with available experimental data and, when available, with existing computational models. This detailed description of neurons allows us to disentangle the effect of each current in the whole-cell dynamics and to drive the design of mutants for experimental validation of *in-silico* findings.

## Supporting information

S1 File The S1 File contains model equations and the tables with the parameters for the modelled currents: SHL1, SHK1, EGL2, IRK, UNC103, KQT1, EXP2, SLO1/2, EGL19, UNC2, CCA1. (PDF)

S1 Fig AVAL KO neurons current clamp simulations. Panels A-D show the comparison of AVAL KO neurons current clamp simulations (colored lines) with the WT simulation (black lines). The simulation consists of 7 current steps from -30 pA to 30 pA with a duration of 1000 ms.
(TIF)

S2 Fig AVAR KO simulations. Panels A-E show the comparison of AVAR KO neurons current clamp simulations (colored lines) with the WT simulation (black lines). The simulation consists of 7 current steps from -30 pA to 30 pA with a duration of 1000 ms.
(TIF)

S3 Fig AIY KO neurons current clamp simulations. Panels A-J show the current clamp simulations of AIY KO neurons (colored lines) compared to the WT simulation (black curve). The simulation protocol consists of 11 current steps ranging from -15 pA to 35 pA with a duration of 5000 ms.
(TIF)

S4 Fig AIY KO neurons voltage clamp simulations. Panels A-J show the voltage clamp simulations of AIY KO neurons (colored lines) compared to the WT simulation (black curve). The voltage clamp protocol consisted of 18 voltage steps from -120 mV to 50 mV with a duration of 500 ms.
(TIF)

S5 Fig RIM KO neurons voltage clamp simulations. RIM KO neurons voltage clamp simulations. Panels A-G show the voltage clamp simulations of RIM KO neurons (colored lines) compared to the WT simulation (black curve).
(TIF)

S6 Fig RIM KO neurons current clamp simulations. Panels A-G show the current clamp simulations of RIM KO neurons (colored lines) compared to the WT simulation (black curve). The simulation protocol consists of 11 current steps ranging from -15 pA to 35 pA with a duration of 5000 ms.
(TIF)

S7 Fig VA5 KO-neurons simulations. **A-F) KO neurons voltage-clamp simulations.** The simulated KO currents (colored lines) are compared with the WT currents represented in black. The simulation protocol consists of 14 voltage steps ranging from -60 mV to 70 mV with a duration of 5000 ms. **G-H) KO neurons current-clamp simulations.** The simulated KO voltage responses (colored lines) are compared with the WT ones (in black). The simulation protocol consists of current steps from -30 pA to 30 pA with 10 pA increments.
(TIF)

S8 Fig VB6 KO-neurons simulations. **A-J) KO neurons voltage-clamp simulations.** The simulated KO currents (colored lines) are compared with the WT currents represented in black. The simulation protocol consists of 14 voltage steps ranging from -60 mV to 70 mV with a duration of 5000 ms. **H-Q) KO neurons current-clamp simulations.** The simulated KO voltage responses (colored lines) are compared with the WT ones represented in black. The simulation protocol consists of current steps from -30 pA to 30 pA with 10 pA increments.
(TIF)

S9 Fig VD5 KO-neurons simulations. **A-J) KO neurons voltage-clamp simulations.** The simulated KO currents (colored lines) are compared with the WT currents represented in black. The simulation protocol consists of 14 voltage steps ranging from -60 mV to 70 mV with a duration of 5000 ms. **H-Q) KO neurons current-clamp simulations.** The simulated KO voltage responses (colored lines) are compared with the WT ones represented in black. The simulation protocol consists of current steps from -30 pA to 30 pA with 10 pA increments.
(TIF)
