# Supplementary material for: Biophysical modeling of the whole-cell dynamics of C. elegans motor and interneurons families
Source: PLoS One. 2024 Mar 29;19(3):e0298105. doi: 10.1371/journal.pone.0298105 (PMC10980225; doi:10.1371/journal.pone.0298105)
Supplement: S1 File — (PDF) [file pone.0298105.s001.pdf]

Supporting information to:

Biophysical modeling of the whole-cell dynamics of C. elegans motor and interneurons families

Martina Nicoletti, Letizia Chiodo, Alessandro Loppini, Qiang Liu, Viola Folli, Giancarlo Ruocco, Simonetta Filippi

Supplementary methods

Experimental data:

The models developed in this work were based on patch-clamp recordings performed in *C. elegans* neurons. The recordings have been performed in the whole-cell configuration with standard electrophysiology protocols for *C. elegans.* Since different groups have performed the experiments, there are some differences in the experimental protocols, mainly in the composition of the bath and pipette solutions and in the components of the experimental set-up. For details about the specific experimental procedures, we refer the reader to the corresponding papers (listed below). In this paper, we rely on experimental recordings obtained in the absence of pharmacological blockers using standard voltage- and current-clamp protocols. In the following, we report for each neuron the reference paper for electrophysiological data and, when available, the link to the raw electrophysiological data used in this work.

- AIY: recordings from [1] (Figure 1 and Figure S1), experimental data available at https://doi.org/10.17632/tngf9w3pgd.1
- RIM: recordings from [1] (Figure 1 and Figure S1), experimental data available at https://doi.org/10.17632/tngf9w3pgd.1
- AVAL and AVAR: recordings from (Figure 8), experimental data available at https://static-content.springer.com/esm/art%3A10.1038%2Fs41467-020-18893-9/MediaObjects/41467_2020_18893_MOESM4_ESM.zip
- VA5: recordings from [2], experimental data available at https://cdn.elifesciences.org/articles/53986/elife-53986-fig4-data1-v2.xlsx
- VB6 and VD5: we extracted the I-V curves from Figure 1 in [3]

Neurons and ionic currents modeling

In this section, we describe in detail the models of the seven neurons and provide the complete set of equations and parameters used in the simulations. The NEURON and Python codes used in this work are available at https://github.com/martinanicoletti92/CelegansInterMotorNeuronsModels.

First, we briefly describe the general equations of the Hodgkin-Huxley model; then, we will summarize the models of the single currents used in this work to model the neurons.

The general equation describing the membrane voltage dynamics of a single-compartment neuron is:

$$C_m \frac{dV}{dt} = -I_{ion} + I_{stim}$$

where, I_{stim} is the external current applied to the neuron to study its responses, I_{ion} is the total ionic current of the cell, including the contribution of potassium, calcium, and leakage currents as follows:

$$I_{AIY} = I_{SHL1} + +I_{SLO1} + I_{KQT1} + I_{EGL19} + I_{NCA} + I_{LEAK}$$

$$I_{RIM} = I_{SHL1} + I_{EGL2} + I_{IRK} + I_{CCA1} + I_{EGL19} + I_{UNC2} + I_{NCA} + I_{LEAK}$$

$$I_{AVAL} = I_{IRK} + I_{EGL19} + I_{NCA} + I_{LEAK}$$

$$I_{AVAR} = I_{IRK} + I_{UNC103} + I_{EGL19} + I_{NCA} + I_{LEAK}$$

$$I_{VA5} = I_{SHK1} + I_{IRK} + I_{SLO2} + I_{EGL19} + I_{NCA} + I_{LEAK}$$

$$I_{VB6} = I_{SHK1} + I_{IRK} + I_{SLO2} + I_{SLO1} + I_{EGL19} + I_{UNC2} + I_{NCA} + I_{LEAK}$$

$$I_{VD5} = I_{SHK1} + I_{IRK} + I_{SLO2} + I_{EGL19} + I_{CCA1} + I_{NCA} + I_{LEAK}$$

The *x-th* ionic current has been modelled according to the Hodgkin-Huxley formalism as follows:

$$I_X = \bar{g}_x \cdot m_x^p \cdot h_x^q \cdot (V - E_{rev})$$

where $\bar{g}_x$ is the maximal conductance listed in Table 3 of the main text, and E_{rev} is the reversal potential of the ionic species: -80 mV for K^+, and 60 mV for Ca^{2+}. m_x^p and h_x^q represent the activation and the inactivation variables, respectively. They are described by the by the following differential equations:

$$\frac{dm_x}{dt} = \frac{m_{x,\infty} - m_x}{\tau_{x,m}}$$

$$\frac{dh_x(V,t)}{dt} = \frac{h_{x,\infty} - h_x}{\tau_{x,h}}.$$

where, $m_{x,\infty}$ and $h_{x,\infty}$ represent the steady state values of the activation and inactivation variables, and $\tau_{x,h}$ and $\tau_{x,m}$ are the activation and inactivation time constants. For the majority of the modelled currents, $m_{x,\infty}$ and $h_{x,\infty}$ are voltage-dependent. However, in the case of BK (SLO1 and SLO2) currents, they also depend on the intracellular calcium. The time constants can be either voltage-dependent or independent, with an additional dependence on intracellular calcium in the case of BK currents. In the following, for each of the ionic currents used in this work, we report the equations for $m_{x,\infty}$, $h_{x,\infty}$, $\tau_{x,h}$ and $\tau_{x,m}$ and we list the corresponding parameters. For further details on how these parameters have been obtained from experimental data and on the models of other currents listed in Table 1 of the main and not used in this work, we refer the reader to [4]. In some cases, the currents were modelled using electrophysiological data obtained through the heterologous expression of the *C. elegans* channels in different cell lines, including CHO, *Xenopus oocytes*, or

HEK cells. In other cases, we used, as reference data, electrophysiological recordings on homologous channels in different organisms.

In addition to the voltage-gated channels, we also model leakage and NCA currents as passive currents with the following equations:

$$I_{NCA} = \bar{g}_{NCA}\,(v - E_{NCA})$$

$$I_{leak} = \bar{g}_{leak}\,(v - E_{rev})$$

The values of $\bar{g}_{NCA}$ and $\bar{g}_{leak}$ are listed in Table 3 of the main text. E_{NCA} was set to 30 mV; while the reversal potential of the leakage current (E_{rev}) has been treated as a free parameter and adjusted in the optimization process (the corresponding values are listed in Table 3 of the main text).

We now describe the models of the ionic currents used in this work. The parameters describing the kinetics of the single currents are the same derived in [4], and were used as fixed parameters in the optimization of the models, during which we only looked for the best set of maximal conductances ($\bar{g}_x$).
The models of SHK1, and intracellular calcium have been updated with respect to [4]. EXP2, KQT1, UNC103, isolated SLO1 and isolated SLO2 currents are new in this work.

Voltage-gated potassium currents

SHL1 (RIM)

$$m_{SHL1,\infty}(V) = \frac{1}{1 + e^{\frac{-(V-V_{0.5})}{k_a}}}$$

$$\tau_{m_{SHL1}}(V) = \frac{a}{e^{\frac{-(V-b)}{c}} + e^{\frac{(V-d)}{\tilde{e}}}} + f$$

$$h^{f}_{SHL1,\infty}(V) = h^{s}_{SHL1,\infty}(V) = \frac{1}{1 + e^{\frac{(V-V_{0.5})}{k_i}}}$$

$$\tau^{f}_{h_{SHL1}}(V) = \tau^{s}_{h_{SHL1}}(V) = \frac{a}{1 + e^{\frac{(V-b)}{c}}} + d$$

$$I_{SHL1} = \overline{g}_{SHL1} \cdot m^{3}_{SHL1} \cdot \left(0.7\,h^{f}_{SHL1} + 0.3\,h^{s}_{SHL1}\right) \cdot (V - E_K)$$

Table S1. Parameters for SHL1 currents.

Parameter		Value	Unit
m_∞	$V_{0.5}$	*-6.8*	mV
	k_a	*14.1*	mV
h_∞	$V_{0.5}$	*-40*	mV
	k_i	*8.3*	mV
τ_m	a	*1.4*	ms
	b	*-17.5*	mV
	c	*12.9*	mV
	d	*-3.7*	mV

	$\tilde{e}$	*6.5*	mV
	f	*0.2*	ms
τ_h^f	a	*53.9*	ms
	b	*-28.2*	mV
	c	*4.9*	mV
	d	*27.3*	ms
τ_h^s	a	*842.2*	ms
	b	*-37.7*	mV
	c	*6.4*	mV
	d	*11.9*	ms
E_K		*-80*	mV

SHK1 (VA5, VB6, VD5)

Model obtained by combining the models in [1], [4] and [1]

$$m_{SHK1,\infty}(V) = \frac{1}{1 + e^{\frac{-(V - V_{0.5})}{k_a}}}$$

$$\tau_{m_{SHK1}}(V) = \frac{a}{e^{\frac{-(V-b)}{c}} + e^{\frac{(V-d)}{\tilde{e}}}} + f$$

$$h_{SHK1,\infty}(V) = \frac{1}{1 + e^{\frac{(V - V_{0.5})}{k_i}}}$$

$$\tau_{h_{SHK1}} = a$$

$$I_{SHK1} = \overline{g}_{SHK1} \cdot m_{SHK1} \cdot h_{SHK1} \cdot (V - E_K)$$

Table S2. Parameters for SHK1 currents.

**Parameter**		**Value**	**Unit**
m_∞	$V_{0.5}$	*2*	mV
	k_a	*10*	mV
h_∞	$V_{0.5}$	*-6.95*	mV
	k_i	*5.8*	mV
τ_m	a	*26.6*	ms
	b	*-33.7*	mV
	c	*15.8*	mV
	d	*-33.7*	mV
	$\tilde{e}$	*15.4*	mV
	f	*2.0*	ms
τ_h		*1400*	ms
E_K		*-80*	mV

EGL2 (RIM)

$$m_{EGL2,\infty}(V) = \frac{1}{1 + e^{\frac{-(V - V_{0.5})}{k_a}}}$$

$$\tau_{m_{EGL2}}(V) = \frac{a}{1 + e^{\frac{(V-b)}{c}}} + d$$

$$I_{\mathrm{EGL2}} = \overline{g}_{EGL2} \cdot m_{EGL2} \cdot (V - \mathrm{E}_K)$$

Table S3. Parameters for EGL2 currents.

Parameter		Value	Unit
m_∞	$V_{0.5}$	*-6.9*	mV
	k_a	*14.9*	mV
τ_m	a	*8.39*	ms
	b	*-122.6*	mV
	c	*13.8*	mV
	d	*4.04*	ms
E_K		*-80*	mV

IRK1/3 (AIY, RIM, AVAL/R, VA5, VB6, VD5)

$$m_{IRK,\infty}(V) = \frac{1}{1 + e^{\frac{(V - V_{0.5})}{k_a}}}$$

$$\tau_{m_{IRK}}(V) = \frac{a}{e^{\frac{-(V-b)}{c}} + e^{\frac{(V-d)}{\tilde{e}}}} + f$$

$$I_{IRK} = \overline{g}_{IRK} \cdot m_{IRK} \cdot (V - E_K)$$

Table S4. Parameters for IRK currents.

Parameter		Value	Unit
m_∞	$V_{0.5}$	*-82*	mV
	k_a	*13*	mV
τ_m	a	*17.1*	ms
	b	*-17.8*	mV
	c	*20.3*	mV
	d	*43.4*	mV
	$\tilde{e}$	*11.2*	mV
	f	*3.8*	ms
E_K		*-80*	mV

UNC103 (AVAR, new in this work)

In the absence of specific experimental data for *C. elegans* UNC-103 we used as reference for the model cardiac delayed rectifier currents [5]

$$m_{UNC103,\infty}(V) = \frac{1}{1 + e^{\frac{-(V-V_{0.5})}{k_a}}}$$

$$\tau^{m}_{UNC103} = \left\{\frac{a}{1 + e^{\frac{(V-b)}{c}}} + d\right\} \cdot \left\{\frac{a}{1 + e^{\frac{-(V-b)}{c}}} + d\right\}$$

$$h_{UNC103,\infty}(V) = \frac{1}{1 + e^{\frac{(V-V_{0.5})}{k_i}}}$$

$$\tau^{h}_{UNC103} = \left\{\frac{a}{1 + e^{\frac{(V-b)}{c}} + d}\right\} \cdot \left\{\frac{a}{1 + e^{\frac{-(V-b)}{c}}} + d\right\}$$

$$I_{UNC103} = \bar{g}_{UNC103} \cdot m \cdot h \cdot (V - E_K)$$

Table S5. Parameters for UNC103 currents.

Parameter		Value	Unit
m_∞	$V_{0.5}$	*-15.1*	mV
	k_a	*7.85*	mV
h_∞	$V_{0.5}$	*-48*	mV
	k_i	*28*	mV
τ_m	a	*87.40*	ms
	b	*-28.33*	mV
	c	*13.10*	mV
	d	*0.26*	ms
τ_h	a	*8.16*	ms
	b	*-25.28*	mV
	c	*29.50*	mV
	d	*0.23*	ms
E_K		*-80*	mV

KQT1 (AIY, new in this work)

We adopted the model of human KCNQ4 currents [6].

$$m_{KQT1,\infty}(V) = \frac{1}{1 + e^{\frac{-(V-V_{0.5})}{k_a}}}$$

$$\tau_{m_{KQT1}}(V) = \frac{a}{1 + e^{\frac{(V-b)}{c}}} + d$$

$$s_{KQT1,\infty}(V) = \frac{a}{1 + e^{\frac{(V-V^{a}_{0.5})}{k^{a}_{s}}}} + \frac{b}{1 + e^{\frac{(V-V_{0.5})}{k^{b}_{s}}}}$$

$$\tau_{s_{KQT1}}(V) = \frac{a}{1 + \left(\frac{V-b}{c}\right)^2} + d$$

$$I_{KQT1} = \overline{g}_{KQT1} \cdot m_{KQT1} \cdot s_{KQT1} \cdot (V - E_K)$$

Table S6. Parameters for KQT1 currents.

Parameter		Value	Unit
m_∞	$V_{0.5}$	*-17.61*	mV
	k_a	*9.58*	mV
s_∞	$V_{0.5}^a$	*-86.84*	mV
	k_s^a	*15.05*	mV
	a	*0.41*	
	$V_{0.5}^b$	*70.13*	mV
	k_s^b	*13.37*	mV
	b	*0.59*	
τ_m	a	*895.9*	ms
	b	*-18.01*	mV
	c	*31.04*	mV
	d	*10*	ms
τ_s	a	*185845*	ms
	b	*39.44*	mV
	c	*7.34*	mV
	d	*1077*	ms
E_K		*-80*	mV

EXP-2 (new in this work)

The model is based on experimental data for *C. elegans* EXP-2 currents: [7]–[9]

$$m_{EXP2\infty}(V) = \frac{1}{1 + e^{\frac{-(V-V_{0.5})}{k_a}}}$$

$$h_{EXP2\infty}(V) = \frac{1}{1 + e^{\frac{(V-V_{0.5})}{k_i}}}$$

$$\tau_{mEXP2,\infty}(V) = \left(\frac{a}{1 + e^{\frac{(V-b)}{c}}} + d\right) \cdot \left(\frac{a}{1 + e^{\frac{(V-b)}{c}}} + d\right)$$

$$\tau_{hEXP2,\infty}(V) = \left(\frac{a}{1 + e^{\frac{(V-b)}{c}}} + d\right) \cdot \left(\frac{a}{1 + e^{\frac{(V-b)}{c}}} + d\right)$$

$$I_{EXP2} = \overline{g}_{EXP2} \cdot m_{EXP2} \cdot h_{EXP2}^2 \cdot (V - E_K)$$

Table S7. Parameters for EXP2 currents.

Parameter		Value	Unit
m_∞	$V_{0.5}$	*-17*	mV
	k_a	*6.5*	mV
h_∞	$V_{0.5}$	*-50*	mV
	k_i	*10*	mV
τ_m	a	*40*	ms
	b	*-20.52*	mV
	c	*4.71*	mV
	d	*2.40*	ms
τ_h	a	*1.28*	ms
	b	*-89.02*	mV
	c	*49.35*	mV
	d	*0.87*	ms
E_K		*-80*	mV

Calcium-regulated potassium currents

SLO1-2 (AIY, VA5, VB6, VD5)

Model of SLO1/2-CaV complex according to [4], [10]. In the following equations, CaV can indicate either EGL19 or UNC2.

$$m_{BK,\infty}(V,Ca) = \frac{m_{CaV} k_o^+ (\alpha + \beta + k_c^-)}{(k_o^+ + k_o^-)(k_c^+ \alpha) + \beta k_c^-}$$

$$\tau_{m_{BK}}(V,Ca) = \frac{\alpha + \beta + k_c^-}{(k_o^+ + k_o^-)(k_c^+ \alpha) + \beta k_c^-}$$

$$\alpha = \frac{m_{CaV,\infty}}{\tau_{m_{CaV}}}$$

$$\beta = \tau_{m_{CaV}}^{-1} - \alpha$$

$$k_{c,o}^- = w^-(V) \cdot f^-(Ca)$$

$$k_{c,o}^+ = w^+(V) \cdot f^+(Ca)$$

$$w^-(V) = w_0^- \cdot e^{-w_{yx}V}$$

$$w^+(V) = w_0^+ \cdot e^{-w_{xy}V}$$

$$f^-(Ca) = \frac{1}{1 + \left(\frac{Ca}{K_{yx}}\right)^{n_{yx}}}$$

$$f^+(Ca) = \frac{1}{1 + \left(\frac{K_{xy}}{Ca}\right)^{n_{xy}}}$$

$$I_{BK} = \overline{g}_{BK} \cdot m_{BK} \cdot h_{CaV} \cdot (V - E_K)$$

Model of isolated SLO1 and SLO2 currents according to [10].

$$m_{iso,BK,j}^{\infty} = \frac{1}{1 + e^{-\frac{V - V_a^{0.5}}{k_a}}}$$

$$V_a^{0.5} = k_a \cdot log\left(\frac{w_0^-}{w_0^+}\right) + log\left(1 + \left(\frac{K_{xy}}{Ca}\right)^{n_{xy}}\right)$$

$$k_a = \frac{1}{w_{yx} - w_{xy}}$$

$$\tau_{m,iso,BK} = \frac{e^{w_{xy}V}}{w_0^+}\left(1 + \left(\frac{K_{xy}}{Ca}\right)^{n_{xy}}\right)\frac{1}{1 + e^{-\frac{V - V_a^{0.5}}{k_a}}}$$

$$I_{BK} = \overline{g}_{BK,\ iso} \cdot m_{BK,iso} \cdot (V - E_K)$$

Table S8. Parameters for SLO1 and SLO2 currents.

Parameter	Value		Unit
	SLO1	SLO2	
w_{yx}	*0.013*	*0.019*	mV^{-1}
w_{xy}	*-0.028*	*-0.024*	mV^{-1}
w_0^-	*3.15*	*0.90*	ms^{-1}
w_0^+	*0.16*	*0.027*	ms^{-1}
K_{xy}	*55.73*	*93.45*	µM
n_{xy}	*1.30*	*1.84*	
K_{yx}	*34.34*	*3294.55*	µM
n_{yx}	10^{-4}	10^{-5}	
E_K	*-80*	*-80*	mV

Voltage-gated calcium currents

EGL-19 (AIY, RIM, AVAL/R, VA5, VB6, VD5)

$$m_{EGL19,\infty}(V) = \frac{1}{1 + e^{\frac{-(V - V_{0.5})}{k_a}}}$$

$$\tau_{m_{EGL19}}(V) = \left[a \sim e^{\left(\frac{V-b}{c}\right)^2}\right] + \left[d \sim e^{-\left(\frac{V-\tilde{e}}{f}\right)^2}\right] + g$$

$$h_{EGL19,\infty}(V) = \left[\frac{a}{1 + e^{\frac{-(V - V_{0.5})}{k_i}}} + b\right] \cdot \left[\frac{c}{1 + e^{\frac{(V - V_{0.5}^b)}{k_i^b}}} + d\right]$$

$$\tau_{h_{EGL19}}(V) = a\left[\frac{b}{1 + e^{\frac{(V-c)}{d}}} + \frac{\tilde{e}}{1 + e^{\frac{(V-f)}{g}}} + h\right]$$

$$I_{EGL19} = \overline{g}_{EGL19} \cdot m_{EGL19} \cdot h_{EGL19} \cdot (V - E_{Ca})$$

Table S9. Parameters for EGL19 currents.

Parameter		Value	Unit
m_∞	$V_{0.5}$	*-5.6*	mV
	k_a	*7.5*	mV
h_∞	$V_{0.5}$	*14.9*	mV
	k_i	*12*	mV
	$V_{0.5}^b$	*-20.5*	mV
	k_i^b	*8.1*	mV
	a	*1.43*	
	b	*0.14*	
	c	*5.96*	
	d	*0.60*	
τ_m	a	*2.9*	ms
	b	*-4.8*	mV
	c	*6.0*	mV
	d	*1.9*	ms
	$\tilde{e}$	*-8.6*	mV
	f	*30*	mV
	g	*2.3*	ms
τ_h	a	*0.4*	
	b	*44.6*	ms
	c	*-33.0*	mV
	d	*5.0*	mV
	$\tilde{e}$	*36.4*	ms
	f	*18.7*	mV
	g	*3.7*	mV
	h	*43.1*	ms
E_{Ca}		*60*	mV

UNC-2 (AIY, RIM, VB6)

$$m_{UNC2,\infty}(V) = \frac{1}{1 + e^{\frac{-(V-V_{0.5})}{k_a}}}$$

$$\tau_{m_{UNC2}}(V) = \frac{a}{e^{\frac{-(V-b)}{c}} + e^{\frac{(V-b)}{d}}} + \tilde{e}$$

$$h_{UNC2,\infty}(V) = \frac{1}{1 + e^{\frac{(V-V_{0.5})}{k_i}}}$$

$$\tau_{h_{UNC2}}(V) = \frac{a}{1 + e^{\frac{-(V-b)}{c}}} + \frac{d}{1 + e^{\frac{(V-\tilde{e})}{f}}}$$

$$I_{UNC2} = \overline{g}_{UNC2} \cdot m_{UNC2} \cdot h_{UNC2} \cdot (V - E_{Ca})$$

Table S10. Parameters for UNC2 currents.

Parameter		Value	Unit
m_∞	$V_{0.5}$	*-37.2*	mV

	k_a	*4.0*	mV
h_∞	$V_{0.5}$	*-77.5*	mV
	k_i	*5.6*	mV
τ_m	a	*1.5*	ms
	b	*-38.2*	mV
	c	*9.1*	mV
	d	*15.4*	mV
	$\tilde{e}$	*0.1*	ms
τ_h	a	*142.5*	ms
	b	*22.9*	mV
	c	*-3.5*	mV
	d	*122.6*	ms
	$\tilde{e}$	*-6.1*	mV
	f	*-3.6*	mV
E_{Ca}		*60*	mV

CCA-1 (RIM, VD5)

$$m_{\mathrm{CCA1},\infty}(V) = \frac{1}{1 + e^{\frac{-(V-V_{0.5})}{k_a}}}$$

$$h_{\mathrm{CCA1},\infty}(V) = \frac{1}{1 + e^{\frac{(V-V_{0.5})}{k_i}}}$$

$$\tau_{m_{\mathrm{CCA1}}}(V) = \frac{a}{1 + e^{\frac{-(V-b)}{c}}} + d$$

$$\tau_{h_{\mathrm{CCA1}}}(V) = \frac{a}{1 + e^{\frac{(V-b)}{c}}} + d$$

$$I_{\mathrm{CCA1}} = \overline{g}_{\mathrm{CCA1}} \cdot m_{\mathrm{CCA1}}^2 \cdot h_{\mathrm{CCA1}} \cdot (V - E_{Ca})$$

Table S11. Parameters for CCA1 currents

**Parameter**		**Value**	**Unit**
m_∞	$V_{0.5}$	*-57.5*	mV
	k_a	*7.6*	mV
h_∞	$V_{0.5}$	*-73.0*	mV
	k_i	*7.0*	mV
τ_m	a	*20.0*	ms
	b	*-92.5*	mV
	c	*21.1*	mV
	d	*0.4*	ms
τ_h	a	*22.4*	ms
	b	*-75.7*	mV
	c	*9.4*	mV
	d	*1.6*	ms
E_{Ca}		*60*	mV

INTRACELLULAR CALCIUM MODEL

In this work we adopted a model of intracellular calcium dynamics developed by Raman *et al.* [11], [12], suitable to be coupled with calcium-regulated potassium channels. Briefly the model describes the intracellular calcium temporal dynamics using the following equation:

$$[Ca^{2+}]_t = [Ca^{2+}]_{t-1} + t\left(-\frac{I_{Ca}}{2FdA} - \beta[Ca^{2+}]_{t-1}\right)$$

where $\beta = 1$ ms^{-1} , is the rate constant, F is the Faraday's constant, d and A are the depth and the area of a 100 nm shell, respectively.

References

[1] Q. Liu, P. B. Kidd, M. Dobosiewicz, and C. I. Bargmann, "C. elegans AWA Olfactory Neurons Fire Calcium-Mediated All-or-None Action Potentials," *Cell*, vol. 175, no. 1, pp. 57-70.e17, 2018, doi: 10.1016/j.cell.2018.08.018.

[2] L.-G. Niu, P. Liu, Z.-W. Wang, and B. Chen, "Slo2 potassium channel function depends on RNA editing-regulated expression of a SCYL1 protein," *Elife*, vol. 9, Apr. 2020, doi: 10.7554/eLife.53986.

[3] P. Liu, B. Chen, and Z. W. Wang, "SLO-2 potassium channel is an important regulator of neurotransmitter release in Caenorhabditis elegans," *Nat Commun*, vol. 5, no. May, pp. 1–12, 2014, doi: 10.1038/ncomms6155.

[4] M. Nicoletti, A. Loppini, L. Chiodo, V. Folli, G. Ruocco, and S. Filippi, "Biophysical modeling of C. elegans neurons: Single ion currents and whole-cell dynamics of AWCon and RMD," *PLoS One*, vol. 14, no. 7, p. e0218738, 2019.

[5] M. C. Sanguinetti, C. Jiang, M. E. Curran, and M. T. Keating, "A mechanistic link between an inherited and an acquird cardiac arrthytmia: HERG encodes the IKr potassium channel," *Cell*, vol. 81, no. 2, pp. 299–307, 1995.

[6] W. C. Tong, R. M. Tribe, R. Smith, and M. J. Taggart, "Computational modeling reveals key contributions of KCNQ and hERG currents to the malleability of uterine action potentials underpinning labor," *PLoS One*, vol. 9, no. 12, 2014, doi: 10.1371/journal.pone.0114034.

[7] M. W. Davis, R. Fleischhauer, J. A. Dent, R. H. Joho, and L. Avery, "A mutation in the C. elegans EXP-2 potassium channel that alters feeding behavior," *Science (1979)*, vol. 286, no. 5449, pp. 2501–2504, 1999.

[8] R. Fleischhauer, M. W. Davis, I. Dzhura, A. Neely, L. Avery, and R. H. Joho, "Ultrafast inactivation causes inward rectification in a voltage-gated K+ channel from Caenorhabditis elegans," *Journal of Neuroscience*, vol. 20, no. 2, pp. 511–520, 2000.

[9] S. Zhang, S. J. Kehl, and D. Fedida, "Modulation of human ether-à-go-go-related K+ (HERG) channel inactivation by Cs+ and K+," *J Physiol*, vol. 548, no. 3, pp. 691–702, 2003.

[10] F. Montefusco, A. Tagliavini, M. Ferrante, and M. G. Pedersen, "Concise whole-cell modeling if BK_Ca-CaV activity controlled by local coupling and stoichiometry," *Biophys J*, vol. 112, pp. 2387–2396, 2017.

[11] I. M. Raman and B. P. Bean, “Inactivation and Recovery of Sodium Currents in Cerebellar Purkinje Neurons: Evidence for Two Mechanisms,” *Biophys J*, vol. 80, no. 2, pp. 729–737, Feb. 2001, doi: 10.1016/S0006-3495(01)76052-3.

[12] Z. M. Khaliq, N. W. Gouwens, and I. M. Raman, “The Contribution of Resurgent Sodium Current to High-Frequency Firing in Purkinje Neurons: An Experimental and Modeling Study,” *The Journal of Neuroscience*, vol. 23, no. 12, pp. 4899–4912, Jun. 2003, doi: 10.1523/JNEUROSCI.23-12-04899.2003.
