# Supplementary figures and images for: Biophysical modeling of the whole-cell dynamics of C. elegans motor and interneurons families
Source: PLoS One. 2024 Mar 29;19(3):e0298105. doi: 10.1371/journal.pone.0298105 (PMC10980225; doi:10.1371/journal.pone.0298105)

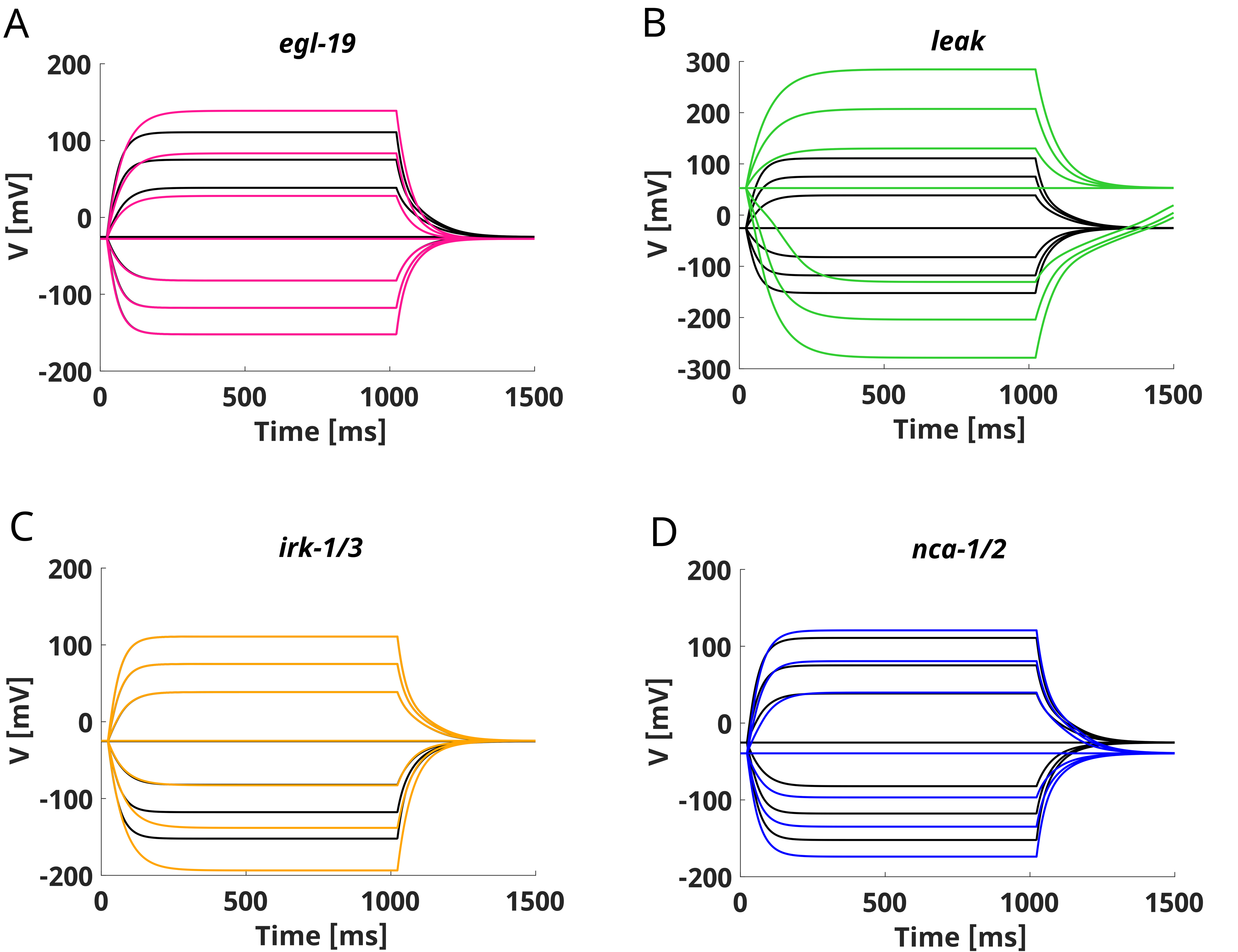

Supplement: S1 Fig — Panels A-D show the comparison of AVAL KO neurons current clamp simulations (colored lines) with the WT simulation (black lines). The simulation consists of 7 current steps from -30 pA to 30 pA with a duration of 1000 ms. (TIF) [file pone.0298105.s002.tif]

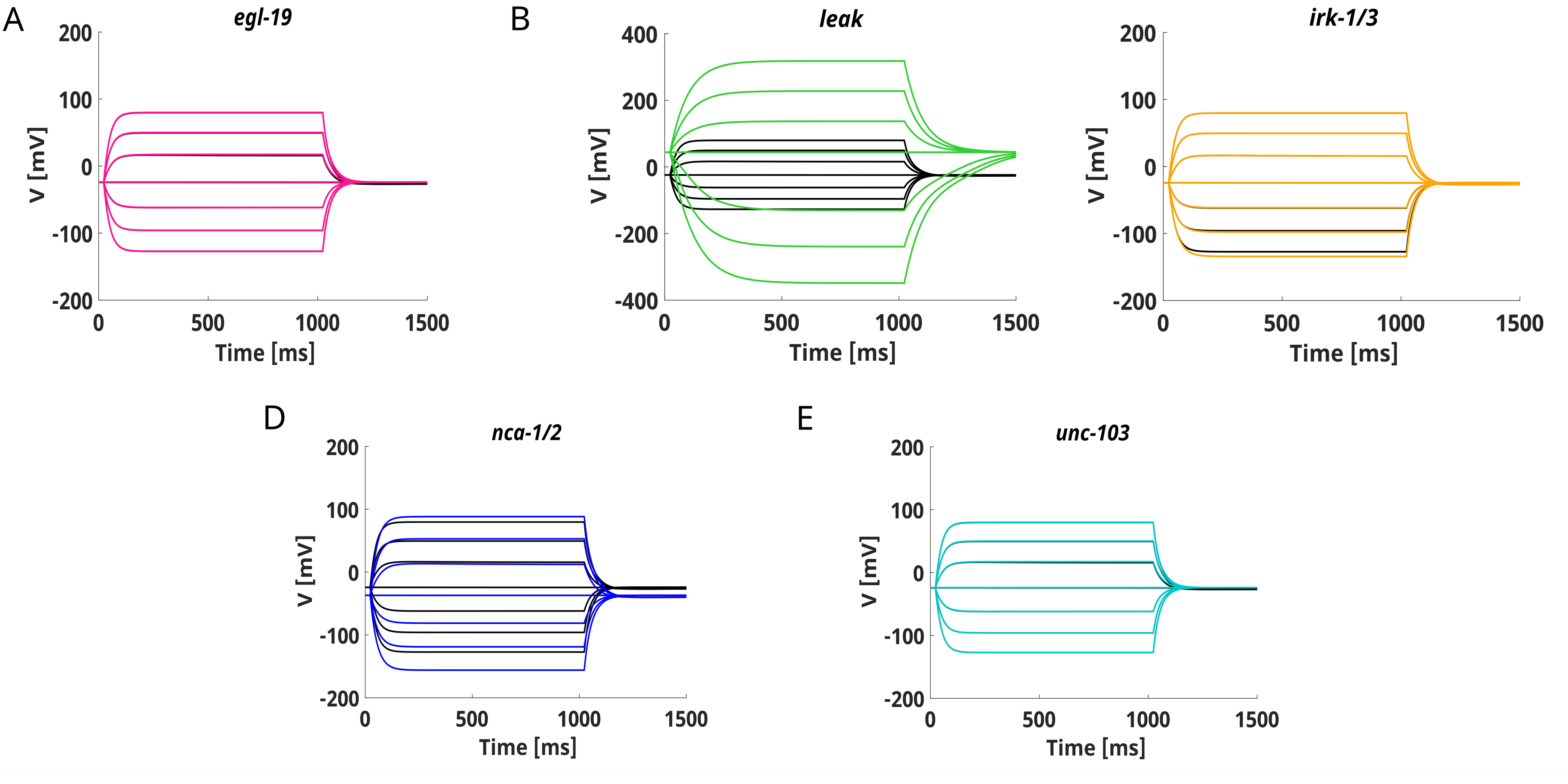

Supplement: S2 Fig — Panels A-E show the comparison of AVAR KO neurons current clamp simulations (colored lines) with the WT simulation (black lines). The simulation consists of 7 current steps from -30 pA to 30 pA with a duration of 1000 ms. (TIF) [file pone.0298105.s003.tif]

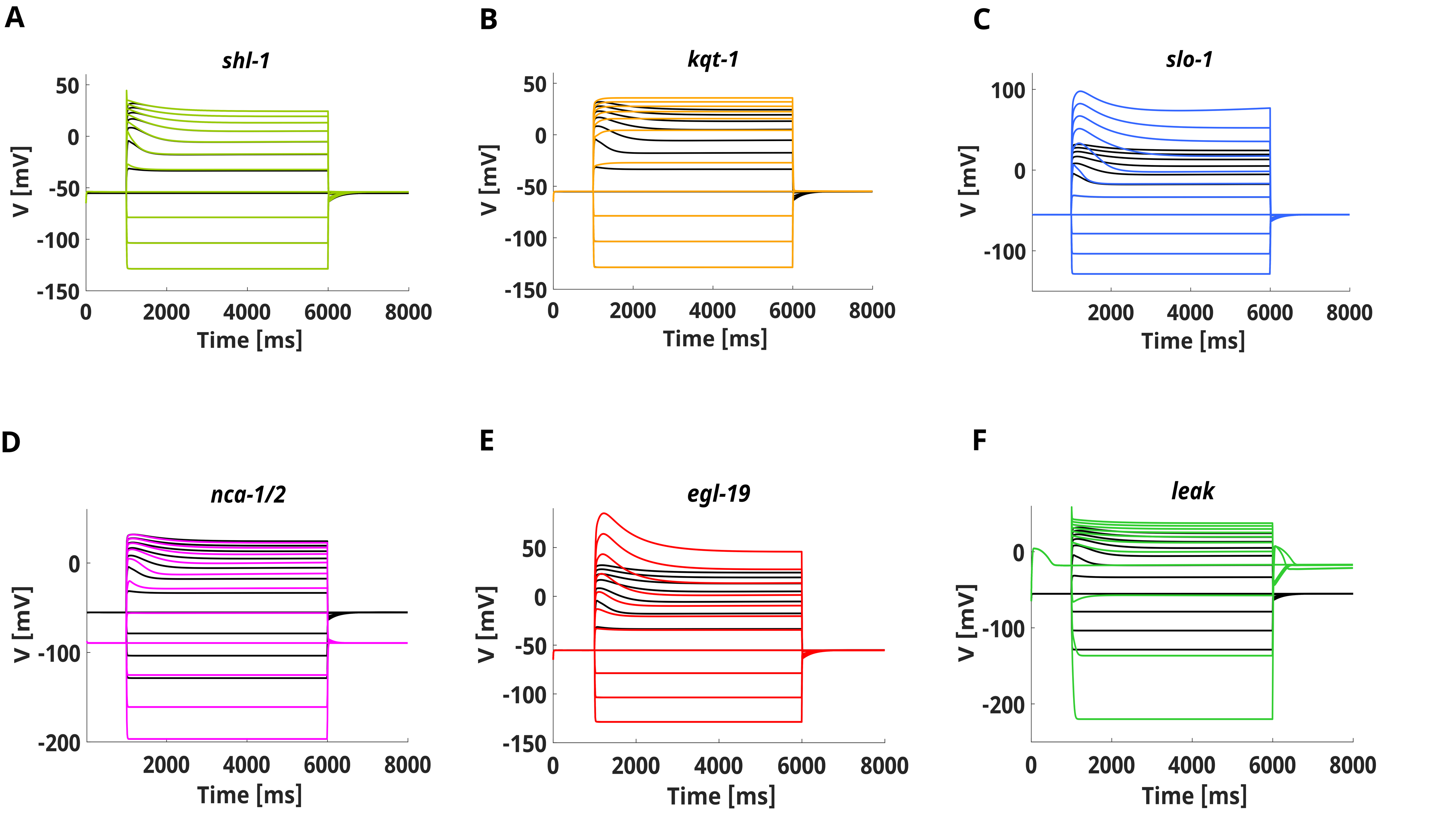

Supplement: S3 Fig — Panels A-J show the current clamp simulations of AIY KO neurons (colored lines) compared to the WT simulation (black curve). The simulation protocol consists of 11 current steps ranging from -15 pA to 35 pA with a duration of 5000 ms. (TIF) [file pone.0298105.s004.tif]

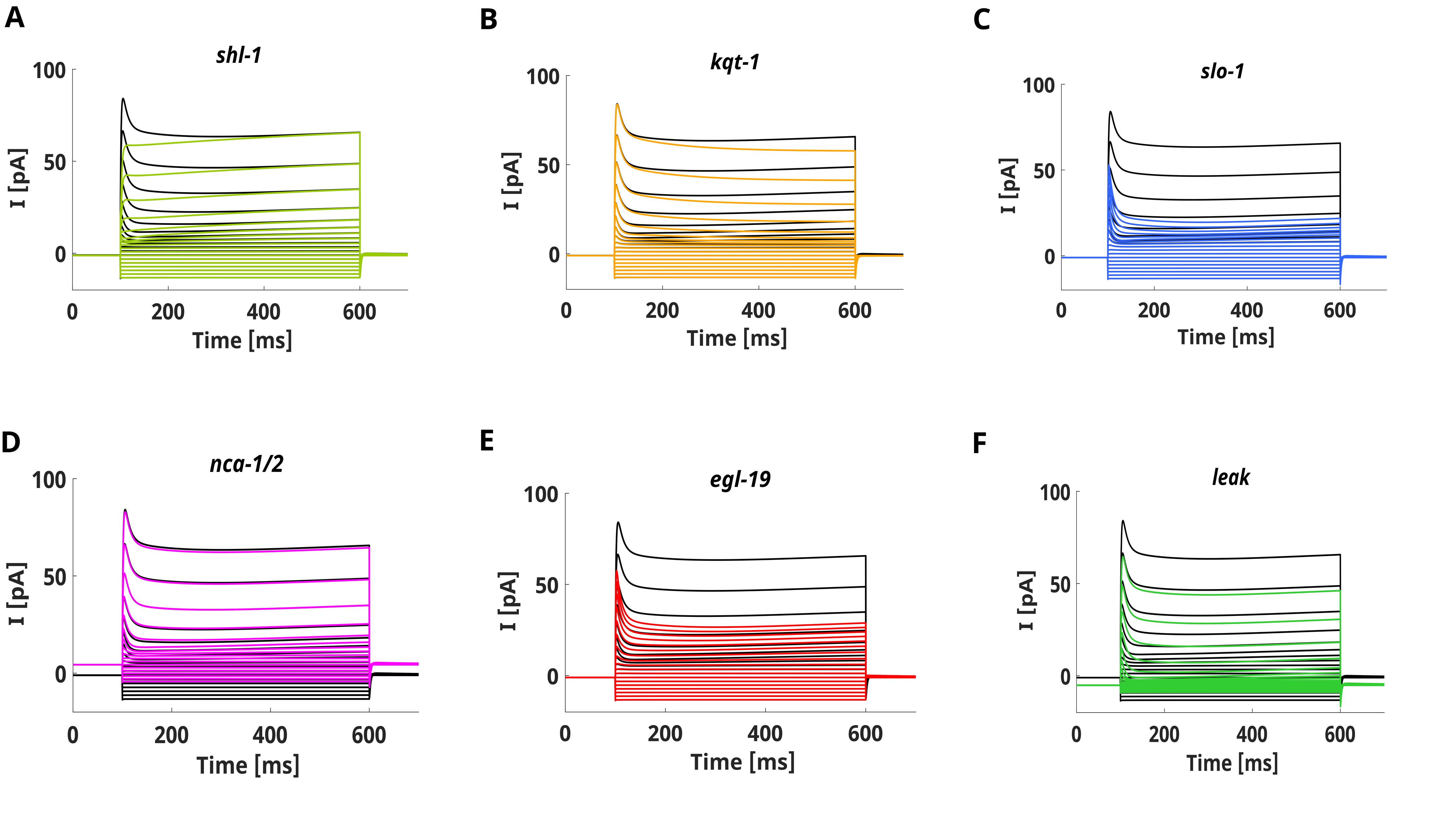

Supplement: S4 Fig — Panels A-J show the voltage clamp simulations of AIY KO neurons (colored lines) compared to the WT simulation (black curve). The voltage clamp protocol consisted of 18 voltage steps from -120 mV to 50 mV with a duration of 500 ms. (TIF) [file pone.0298105.s005.tif]

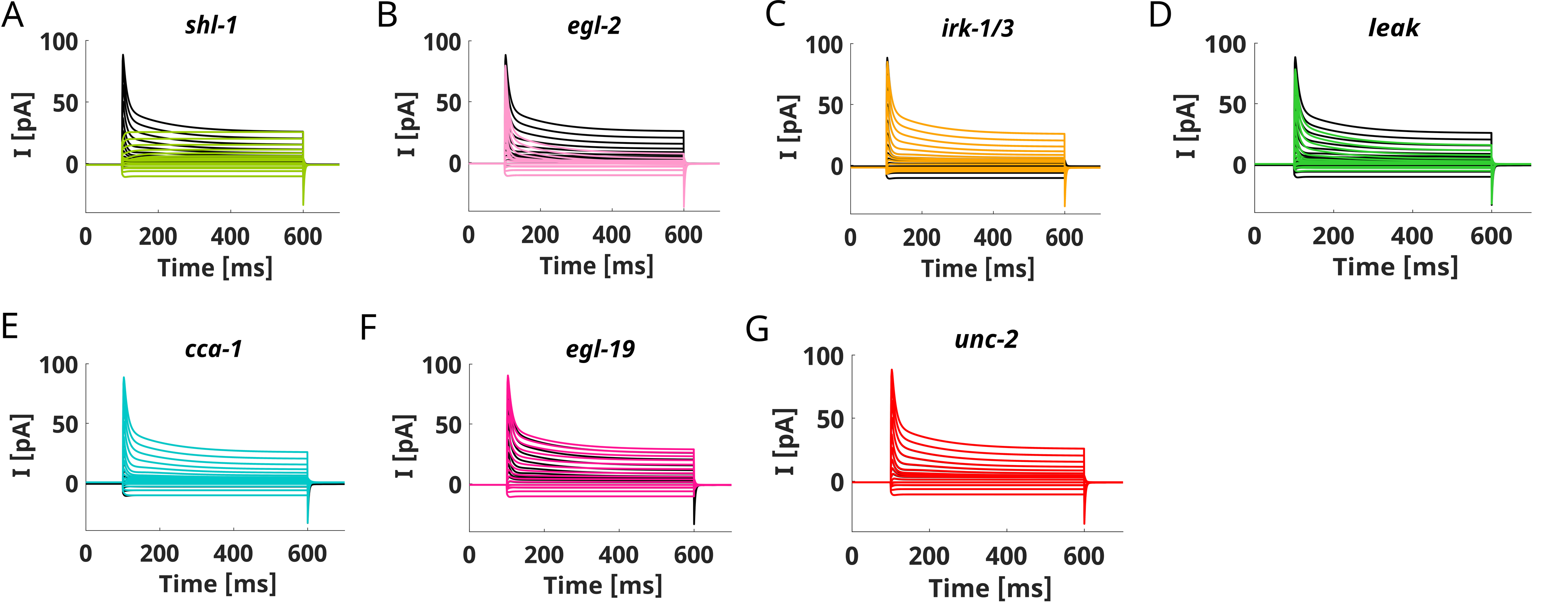

Supplement: S5 Fig — RIM KO neurons voltage clamp simulations. Panels A-G show the voltage clamp simulations of RIM KO neurons (colored lines) compared to the WT simulation (black curve). (TIF) [file pone.0298105.s006.tif]

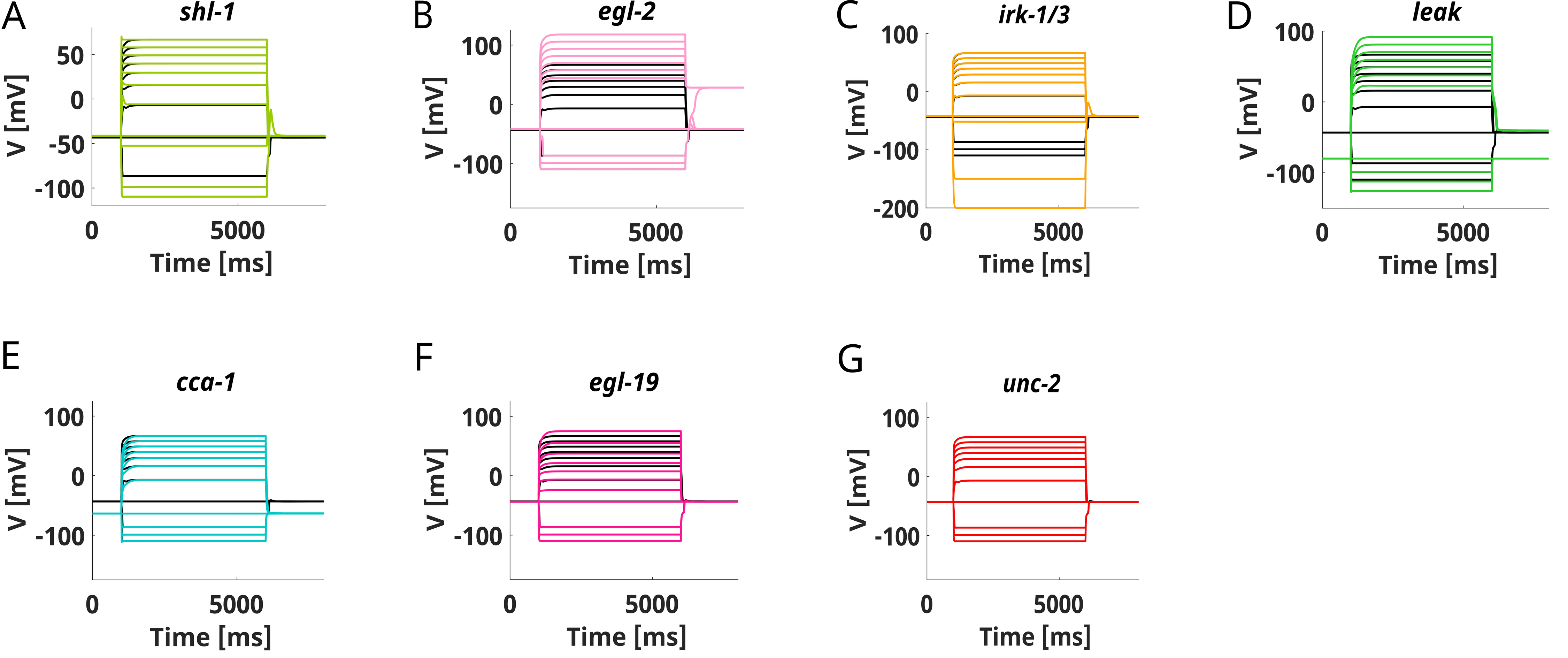

Supplement: S6 Fig — Panels A-G show the current clamp simulations of RIM KO neurons (colored lines) compared to the WT simulation (black curve). The simulation protocol consists of 11 current steps ranging from -15 pA to 35 pA with a duration of 5000 ms. (TIF) [file pone.0298105.s007.tif]

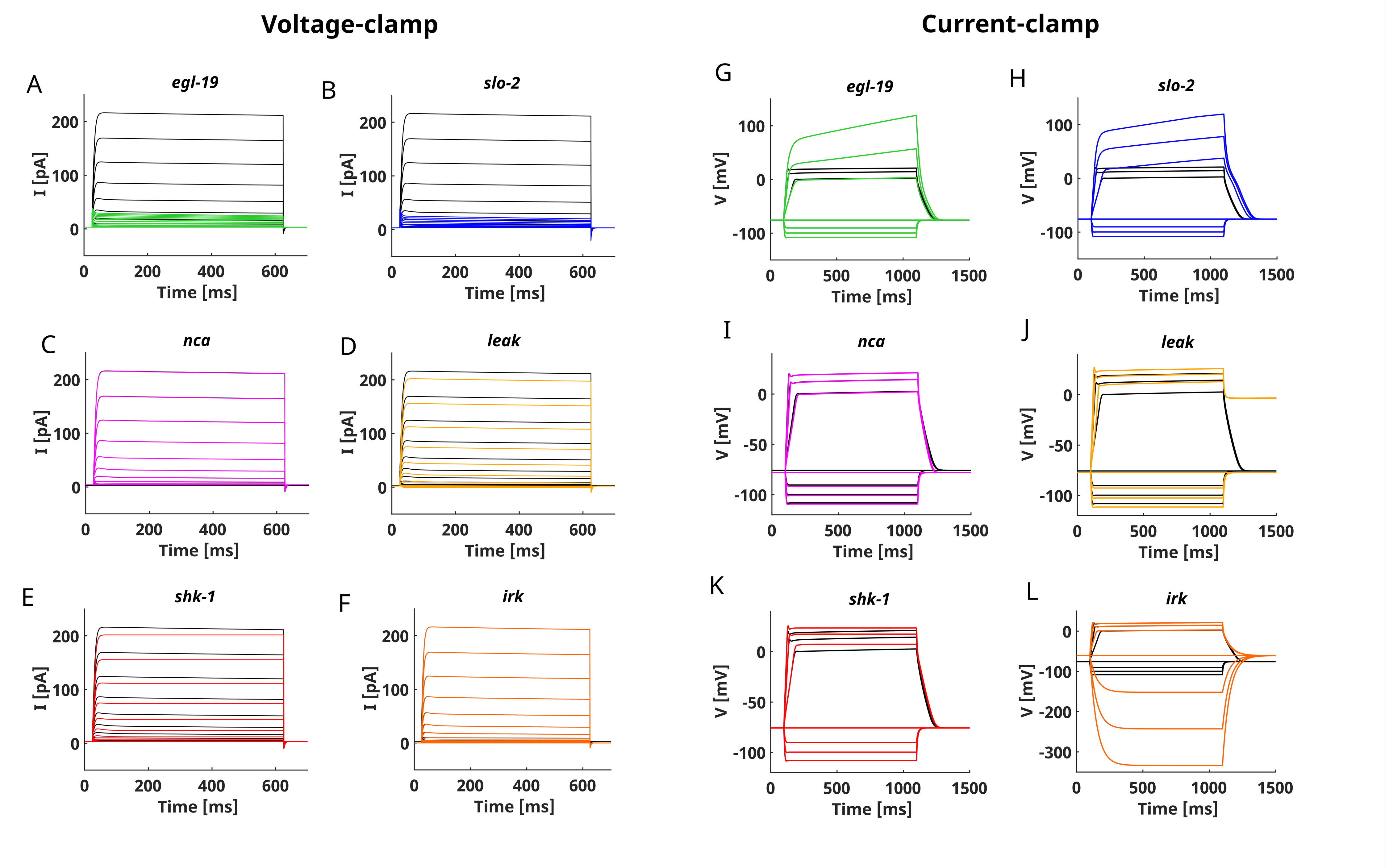

Supplement: S7 Fig — A-F) KO neurons voltage-clamp simulations. The simulated KO currents (colored lines) are compared with the WT currents represented in black. The simulation protocol consists of 14 voltage steps ranging from -60 mV to 70 mV with a duration of 5000 ms. G-H) KO neurons current-clamp simulations. The simulated KO voltage responses (colored lines) are compared with the WT ones (in black). The simulation protocol consists of current steps from -30 pA to 30 pA with 10 pA increments. (TIF) [file pone.0298105.s008.tif]

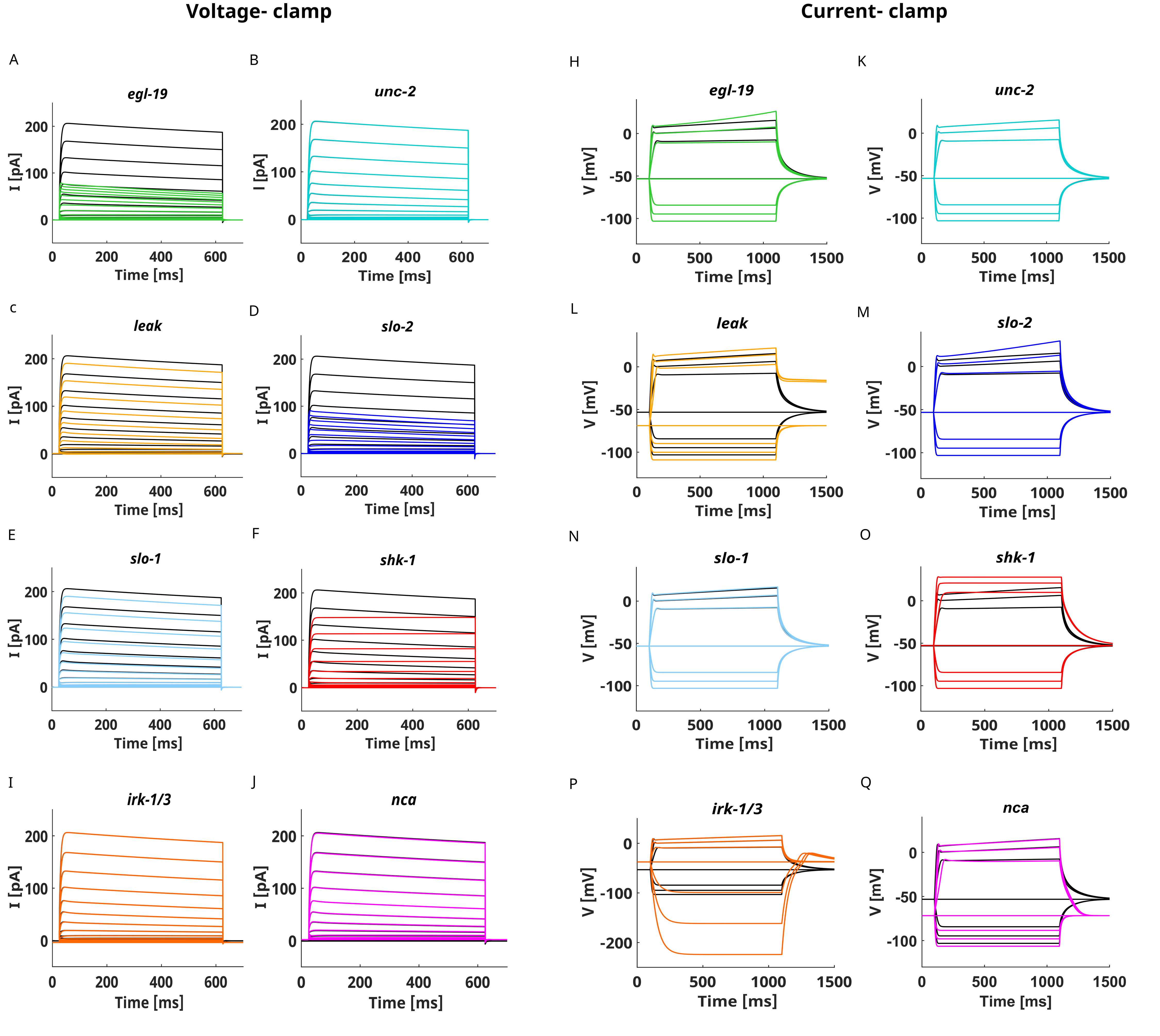

Supplement: S8 Fig — A-J) KO neurons voltage-clamp simulations. The simulated KO currents (colored lines) are compared with the WT currents represented in black. The simulation protocol consists of 14 voltage steps ranging from -60 mV to 70 mV with a duration of 5000 ms. H-Q) KO neurons current-clamp simulations. The simulated KO voltage responses (colored lines) are compared with the WT ones represented in black. The simulation protocol consists of current steps from -30 pA to 30 pA with 10 pA increments. (TIF) [file pone.0298105.s009.tif]

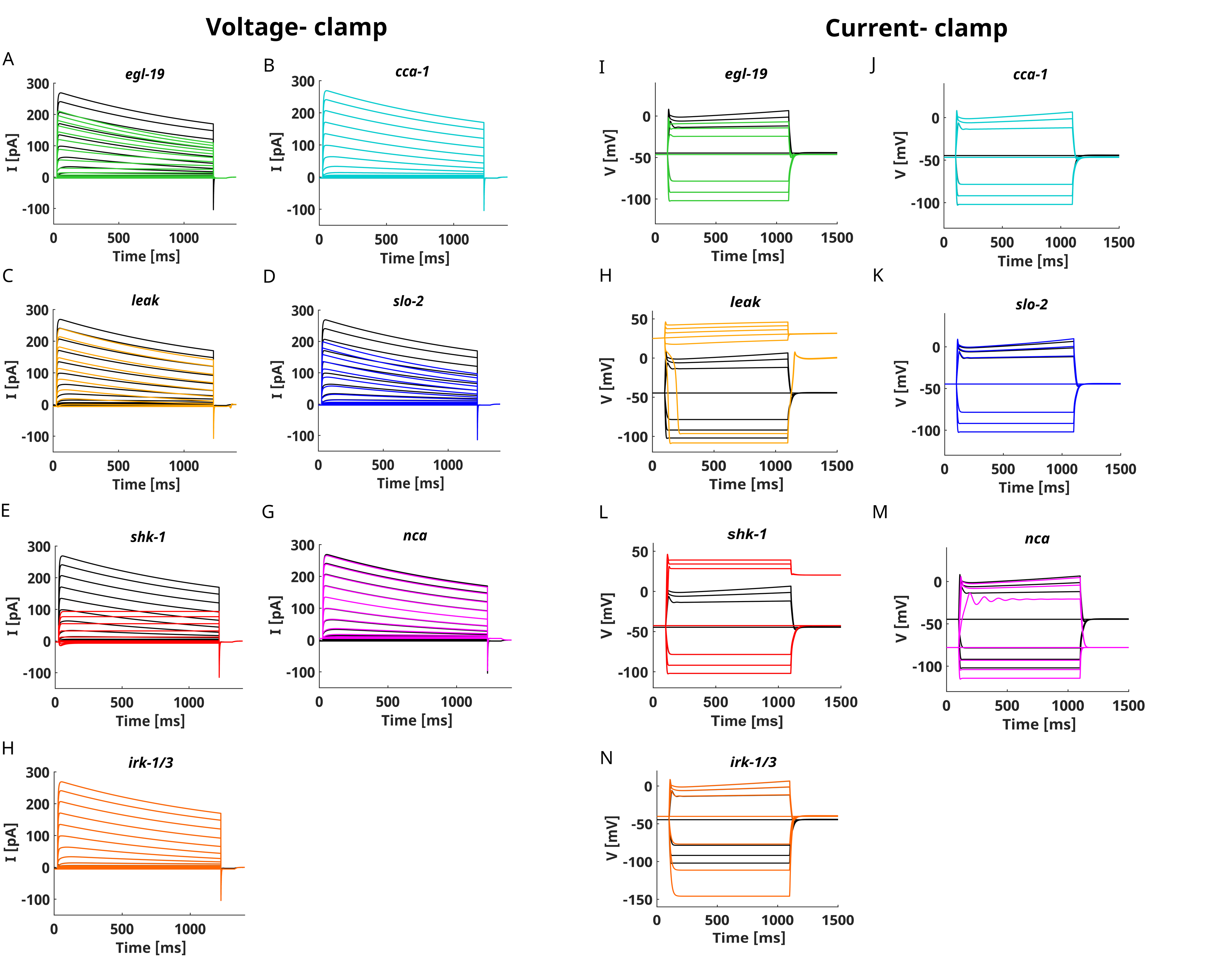

Supplement: S9 Fig — A-J) KO neurons voltage-clamp simulations. The simulated KO currents (colored lines) are compared with the WT currents represented in black. The simulation protocol consists of 14 voltage steps ranging from -60 mV to 70 mV with a duration of 5000 ms. H-Q) KO neurons current-clamp simulations. The simulated KO voltage responses (colored lines) are compared with the WT ones represented in black. The simulation protocol consists of current steps from -30 pA to 30 pA with 10 pA increments. (TIF) [file pone.0298105.s010.tif]
